# Technical progress and clinical application of distal nerve transfers

**DOI:** 10.3389/fsurg.2026.1789006

**Published:** 2026-07-21

**Authors:** Lukas Rasulić, Andrija Savić, Aleksa Mićić, Jovan Grujić, Milan Lepić, Vojin Kovačević

**Affiliations:** 1 University of Belgrade, Faculty of Medicine, Belgrade, Serbia; 2 University Clinical Center of Serbia, Neurosurgery Clinic, Belgrade, Serbia; 3 University Clinical Center of Serbia, Neurosurgery Clinic, Kragujevac, Serbia; 4 University of Defence, Faculty of Medicine, Belgrade, Serbia; 5 Military Medical Academy, Neurosurgery Clinic, Belgrade, Serbia; 6 University of Kragujevac, Faculty of Medical Sciences, Department of Surgery, Kragujevac, Serbia

**Keywords:** brachial plexus, distal nerve transfer, nerve regeneration, peripheral nerve injury, spinal cord injury, surgical outcomes

## Abstract

**Introduction:**

Distal nerve transfer surgery has emerged as a transformative strategy in the treatment of peripheral nerve injuries. However, substantial heterogeneity in injury patterns, donor selection, surgical techniques, and outcome measures has produced conflicting evidence and limited consensus regarding optimal reconstructive approaches. To address these limitations, we propose a concept of four-level decision-making framework integrated with a donor feasibility triad consisting of availability, suitability, and acceptability.

**Methods:**

This paper integrates the author's surgical experience with current literature and conceptualizes the decision-making process through the proposed framework. No formal systematic review or original data collection was performed. Three testable predictions were derived and assessed using evidence from systematic reviews, meta-analyses, and large clinical series.

**Results:**

The reviewed literature demonstrated substantial heterogeneity and frequent contradictions regarding donor selection, reconstructive strategies, and technical approaches. There was also substantial heterogeneity in the evaluation of the three predictions, as they were primarily qualitative constructs without standardized quantitative thresholds or uniform assessment criteria. Prediction 1, proposing a worse outcome prognosis with an increase in lesion severity, was consistently supported. Prediction 2, proposing superior outcomes with feasibility triad-compliant donor selection, remains insufficiently evaluated. Prediction 3, proposing that technical modifications and adjunctive modalities cannot fully compensate for poor donor feasibility, was supported.

**Discussion:**

The framework does not prescribe a fixed algorithm but provides a structured language to articulate why a donor choice was made, what constraints existed, and where uncertainties lie, enabling meaningful comparison across heterogeneous clinical scenarios. Prospective validation of the predictions and standardized outcome reporting are needed to improve interpretability and reproducibility across studies.

## 1 Introduction

Distal nerve transfer surgery has emerged over the past decade as a major advancement in peripheral nerve repair. By rerouting expendable donor fascicles directly to denervated recipient nerves near their target organs, these transfers substantially reduce regenerative distance, accelerate reinnervation, and improve the likelihood of timely and clinically meaningful recovery. Initially experimental, these techniques have evolved into standard practice across multiple clinical contexts, demonstrating superior outcomes in selected indications (1, 2).

Despite significant advances, major discrepancies persist regarding the optimal reconstructive strategy. First, the choice between nerve grafting, nerve transfer, and tendon transfer techniques remains debated. Indication of distal nerve transfer surgery for surgery and selection of strategy varies among the surgeons, and there is no universal consensus. Comparative studies are scarce, and available evidence often consists of small, single-center series with heterogeneous outcome measures (3–6).

To address these discrepancies, we propose a four-level decision-making framework grounded in the technique feasibility triad (availability, suitability, acceptability). By integrating current literature with the authors' extensive surgical experience (7–22), this article provides a practical, evidence-informed approach for conceptualizing available reconstructive strategies, presenting a testable hypothesis that outcomes of distal nerve transfers can be optimized through systematic application of this framework across all levels.

## 2 Methods

This article presents a conceptual four-level decision-making framework for distal nerve transfer surgery based on the authors' collective surgical experience, established biological principles of nerve regeneration, and clinical problem-solving patterns. No formal systematic review or original data collection was performed. For each anatomically feasible donor nerve relevant to a given target function, we reviewed the literature to identify all described options, with emphasis on the donors most feasible and most commonly reported. We then applied the framework sequentially, through its four levels to organize reconstructive strategies.

To assess our three testable predictions, we focused on evidence from systematic reviews and meta-analyses where available, supplemented by large clinical series. The framework and discussion are limited to distal nerve transfers in adult patients with traumatic peripheral nerve injuries, brachial plexus injuries, and spinal cord injury; we did not address contralateral nerve transfers, cranial nerve transfers, the pediatric population, or applications in stroke or autoimmune neurodegenerative diseases. The original figures (2–12) were created by the authors to illustrate previously unpublished technical concepts, and artificial intelligence tools (ChatGPT, DeepSeek) were used exclusively for language refinement and structural organization of the manuscript.

## 3 A four-level decision-making framework

Distal nerve transfer reconstruction requires a sequence of decisions that extends from the initial clinical indication to the final technical execution. These decisions are constrained by individual patient characteristics, the severity and pattern of injury, and the feasibility of available reconstructive techniques. Together, these factors determine the prioritization of functional restoration and guide the selection of appropriate recipient and donor nerves to achieve meaningful functional recovery (23, 24).

**Figure 1:**
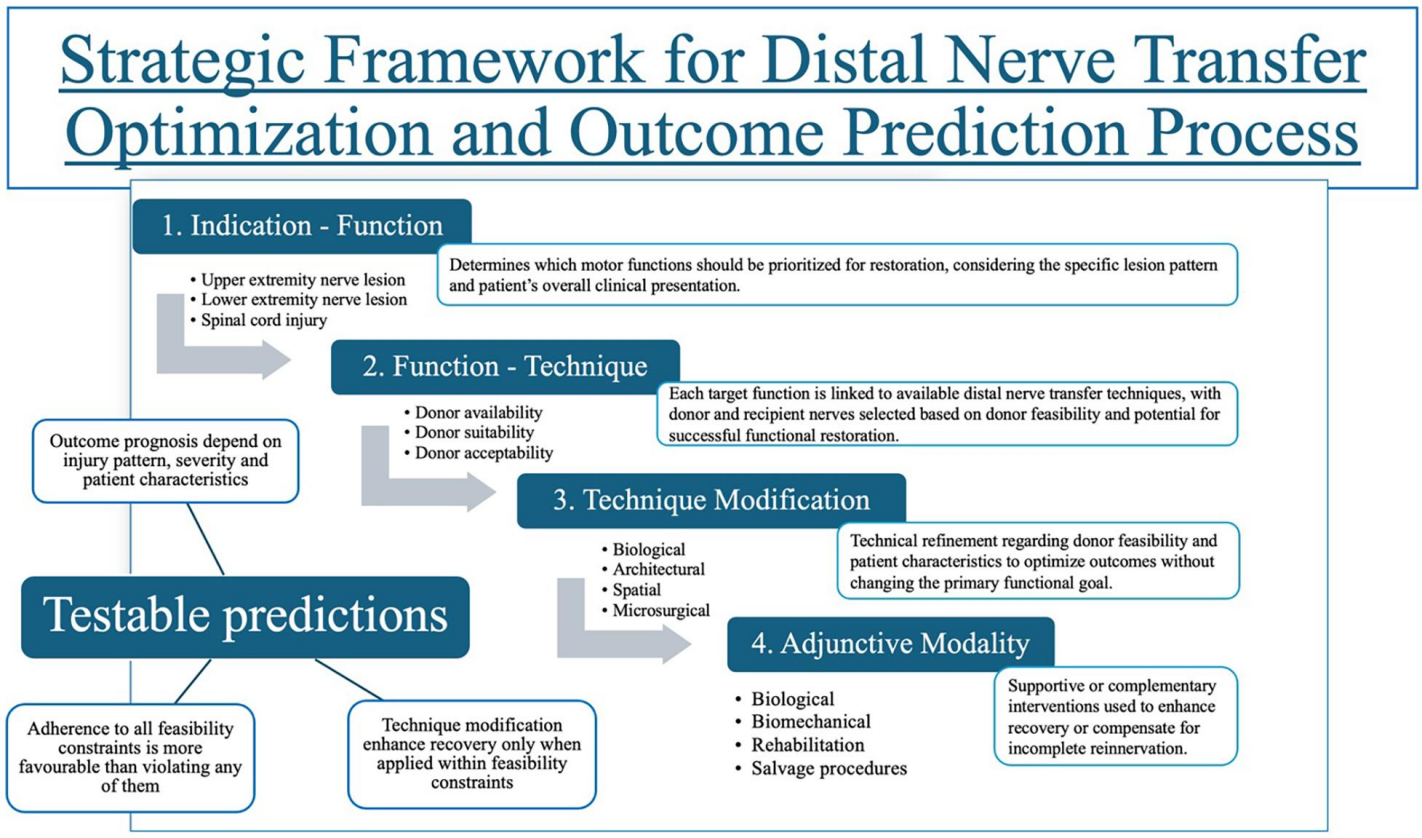
Four-level strategic framework for optimization of decision-making and outcome prediction process in distal nerve transfer surgery.

To conceptualize this process, we propose a four-level decision-making framework that generates testable predictions linking lesion extent, priorities for functional restoration, and reconstruction feasibility. (Figure 1) By integrating these elements, the framework provides evidence-based expectations for surgical outcomes and suggests that adherence to these decision levels may optimize surgical strategy and enhance the prediction of functional recovery.

### 3.1 Reconstruction technique feasibility

The feasibility of a given reconstructive technique is assessed through availability, suitability, and acceptability. These factors may already inform clinical planning during the indication stage, while continuously refined intraoperatively, when direct assessment of injury extent, severity, and individual anatomical variations allows a more precise evaluation.

- **Availability** refers to whether a potential donor nerve is present, functional, and surgically accessible. This includes anatomical presence, preserved function, reach within the operative field, and possibility of exposure considering body habitus and scarring due to prior surgery, radiation, or local pathology. A donor nerve that is anatomically present but functionally compromised by injury, denervation, or previous intervention may therefore be considered unfeasible for transfer (25).
- **Suitability** refers to whether an available donor nerve possesses the structural and biological characteristics necessary for effective reinnervation. Relevant factors include nerve diameter compatibility, motor to sensory fascicular ratio, axon count, and appropriate length to achieve a tension-free coaptation. Suitability also incorporates the capacity for neural plasticity, recognizing that functional recovery depends not only on anatomical matching but also on the ability of the peripheral and central nervous systems to reorganize following the transfer (26).
- **Acceptability** reflects the clinical permissibility of using a given donor nerve. It incorporates donor redundancy—the presence of compensatory neural pathways that allow preservation of the original function despite partial donor sacrifice—and anticipated donor-site morbidity, referring to the expected functional deficit in the donor territory following nerve harvest. Acceptability, therefore, requires balancing the potential functional gain of the transfer against the anticipated loss of donor function in the individual patient (27).

### 3.2 Framework overview

Within the constraints defined by the feasibility triad, decision-making proceeds through four sequential levels:

#### 3.2.1 Level 1 (indication—function)

Indication for surgery and selection of target functions are determined by the injury pattern and resulting neurological deficits. Priorities for functional restoration are guided by established reconstructive hierarchies in the literature, while remaining closely tailored to what is surgically feasible given the pattern and extent of injury (28–30).

#### 3.2.2 Level 2 (function—technique)

Once priorities for functional restoration are defined, target functions are matched with appropriate distal nerve transfer options. The final donor-recipient combination is selected based on established practice patterns, while remaining adaptable to intraoperative assessment of feasibility, influenced by the extent of tissue injury and individual anatomical variations (31–34).

#### 3.2.3 Level 3 (technique modification)

To enhance feasibility, the chosen transfer technique can be refined through biological, architectural, spatial, or microsurgical modifications without altering the primary functional indication (35, 36).

#### 3.2.4 Level 4 (adjunctive modalities)

Additional interventions are integrated to optimize the chosen reconstructive strategy, support regeneration, prevent dennervation or compensate insufficient recovery (37–39).

At all levels, availability, suitability, and acceptability continuously guide decisions. Any technique violating feasibility should not be prioritized or performed, regardless of theoretical attractiveness.

### 3.3 Testable predictions

The four-level decision framework, with feasibility placed at its foundation, generates explicit testable predictions:

#### 3.3.1 Lesion severity and donor constraint

With increasing nerve lesion severity, a higher number of targeted functions, and more limited donor nerve availability, the overall functional outcomes (MRC grade), patient satisfaction, and quality of life will be progressively lower, even when surgical techniques are optimized. This reflects the deteriorating donor-to-target ratio as injury extent expands.

#### 3.3.2 Feasibility triad compliance

Donor–recipient pairings satisfying all three triad criteria, availability, suitability, and acceptability—are predicted to yield superior functional outcomes compared to pairings that violate one or more criteria. Transfers attempted without satisfying these feasibility constraints are predicted to be less reliable, even if the anatomical match appears theoretically sound.

#### 3.3.3 Effectiveness of technique modifications and adjunctive modalities

The use of technical refinements and adjunctive strategies may improve overall outcomes. However, they cannot compensate for inadequate donor nerve selection or the absence of a suitable donor.

## 4 Clinical implementation of framework

This section illustrates the application of the four-level framework in clinical practice. Implementation is structured sequentially, primarily by determining the indications and selecting functional targets for restoration, followed by choosing the appropriate reconstruction technique, and finally, by considering technical modifications and adjunctive modalities to optimize outcomes.

### 4.1 Implementation at the indication-function level

At the indication–function level, clinical implementation begins with a thorough evaluation of the patient's functional deficits and identification of the anatomical targets for restoration. In isolated peripheral nerve injuries, reconstructive strategies focus on restoring the specific functions lost due to the injured nerves. Challenges arise in extensive lesions with preoperatively unrecognized donor morbidity, multiple nerve injuries, such as in BP injuries, due to increasing competition among functional priorities and the availability of feasible donor options (40, 41).

While no universal consensus defines the exact sequence of functional recovery (42), surgical strategies typically prioritize movements that are essential for daily activities and have the greatest impact on patient independence and quality of life (43). Selection of these critical functions is informed by the patient's overall potential for recovery, the severity and type of nerve injury, technique feasibility, and the timing of intervention, ensuring that reconstructive efforts are both meaningful and strategically focused (44).

#### 4.1.1 Isolated peripheral nerve injuries

In isolated peripheral nerve injuries of the upper extremity, selecting the priority function is usually achievable, and technical reconstruction is feasible since adjacent intact nerves are viable and accessible within the same surgical approach (Table 1). In the lower extremity, functional targets of priority are less established than in the upper extremity, with some sensory transfers emphasized as potential targets. However, the literature is scarce, and outcomes remain difficult to compare due to variable metrics and follow-up (45, 46).

**Table 1:** Functional targets for restoration in isolated peripheral nerve injuries.

Indication category	Specific lesion pattern	Functions targeted for restoration
***Isolated nerve injuries—*** *upper extremity*	Musculocutaneous nerve Axillary nerve Suprascapular nerve Long thoracic nerve	Elbow flexion Shoulder abduction; Shoulder external rotation; Scapular rotation
	Radial nerve (high)	Elbow extension Wrist extension; Finger extension Thumb abduction
	Median nerve (high)	Finger flexion Wrist flexion Thumb opposition Tactile sensitivity
	Ulnar nerve (high)	Hand intrinsic function
**Isolated nerve injuries—**lower extremity	Common peroneal nerve	Ankle dorsiflexion; Toe extension
	Deep peroneal nerve	Ankle dorsiflexion
	Tibial nerve^a^	Plantarflexion; Push-off stability
	Femoral nerve	Knee extension
	Obturator nerve^a^	Hip adduction

a Rarely reported in the literature, highly selected cases.

**Table 2:** Functional targets for restoration in brachial plexus (BP) palsy, stratified by lesion pattern.

Indication category	Specific lesion pattern	Functions targeted for restoration
Brachial plexus palsy (BP)	Upper palsy C5-C6 roots Upper BP trunk Lateral BP fascicle	Elbow flexion; Shoulder abduction; Shoulder external rotation
	Extended upper palsy C5-C6-C7 roots Upper/middle BP trunks Lateral/inferior BP fascicles	Elbow flexion; Shoulder abduction; Shoulder external rotation; Elbow extension Wrist extension Finger extension
	Lower palsy C8-T1 roots Lower BP trunk Medial BP fascicle	Wrist flexion Finger flexion Wrist extension Finger extension
	Complete palsy C5-T1 roots All trunks All fascicles	Elbow flexion; Shoulder abduction; Shoulder external rotation

#### 4.1.2 Combined peripheral nerve injuries

When two or more peripheral nerves are injured simultaneously, reconstructive options become inherently constrained, as commonly used donor nerves may be compromised by denervation. Across all such patterns, meticulous preoperative evaluation of any preserved donor function is indispensable, and management often departs from standard donor-recipient schemes (47–51).

#### 4.1.3 Brachial Plexus injuries

Reconstructive priorities in BP injuries depend on the extent and level of neural involvement, impacting the number of functional targets and the available donor nerves, with achievable outcomes generally diminishing as the severity of the injury increases.

While upper BP palsy (C5-C6) generally preserves a favorable donor-to-target ratio that permits established reconstructive strategies for elbow flexion, shoulder abduction, and external rotation, extended upper BP palsy (C5-C7) introduces additional deficits involving elbow, wrist, and finger extension, thereby increasing reconstructive demands while simultaneously reducing donor availability. This necessitates selective prioritization based on lesion severity and donor feasibility.

In lower BP palsy (C8-T1), reconstructive strategy is largely restricted to selective restoration of distal hand function because fine motor control and tactile sensation require a high axonal load relative to the limited donor pool. In complete BP palsy (C5-T1), the disparity between global functional loss and available donors becomes critical, restricting reconstruction primarily to priority functions such as elbow flexion, followed secondarily by shoulder abduction or external rotation (52–57).

#### 4.1.4 Spinal cord injury

Nerve transfers in spinal cord injury (SCI) represent an emerging indication-driven extension of distal nerve transfer principles, using preserved peripheral motor pathways above the level of injury to restore function below it. In contrast to traumatic peripheral nerve lesions, the target nerves and muscles in SCI remain anatomically intact but are functionally disconnected from supraspinal control. SCI is primarily an upper motor neuron lesion that preserves the lower motor neuron and neuromuscular junction, while BP injury is a lower motor neuron lesion characterized by direct peripheral axonal disruption (58).

Preservation of the LMN in SCI allows target muscles to remain viable for prolonged periods, contrary to the BP injuries, which result in rapid denervation, motor endplate degeneration, and progressive muscle atrophy, leading surgeons to prioritize restoration of proximal, coarse motor functions such as elbow flexion and shoulder abduction, which require shorter reinnervation distances and lower donor axon counts (59–61). Therefore, the selection of target functions and donors in SCI is based on its level, extent, and severity (62).

In high cervical SCI (C3-C4), restoration of diaphragmatic function by reinnervating PHN with ICNs or SAN is the only nerve transfer option (63–68), while with a lower injury level, the rate of donor availability increases. The elbow, wrist, and finger extension, followed by finger flexion, are primary targets for restoration in these patients (69, 70).

Nerve transfers for thoracic and lumbar spinal cord injury are a rapidly developing frontier, but their application differs significantly from the more established protocols for cervical injuries. For individuals with injuries at these levels, the primary goals are to restore mobility, continence, and sexual function to achieve greater independence (71).

### 4.2 Implementation at the function-technique level

At the function-technique level, each target function corresponds to one or more established donor nerve options described in the literature, and technique selection generally follows these standard donor–recipient pairings considered most feasible, (Table 2) and therefore most commonly performed and reported, while there is no universal consensus (72). Deviation from the primary donor strategy may occur when patient-specific factors, particularly variations in injury severity or local anatomical constraints, limit donor feasibility. In such circumstances, additional or alternative donor nerves may be employed to achieve the same functional objective (73).

#### 4.2.1 Shoulder function

Restoration of shoulder function after peripheral nerve injury addresses three main deficits: impaired abduction, limited external rotation, and reduced scapular rotation. Shoulder abduction and external rotation depend on coordinated, synergistic activation of the AXN and SSN, modulated by scapulothoracic stability, which is governed by the SAN and LTN. When either is compromised, even anatomically successful AXN or SSN reinnervation may translate into suboptimal global shoulder function due to loss of the proximal scapular base required for effective glenohumeral motion. Therefore, the donor and recipient nerves are selected according to the specific injury pattern, the amaount of available motor donors, and the technical feasibility of the transfer technique, to restore balanced, coordinated shoulder mechanics rather than isolated muscle actions.

**Figure 2:**
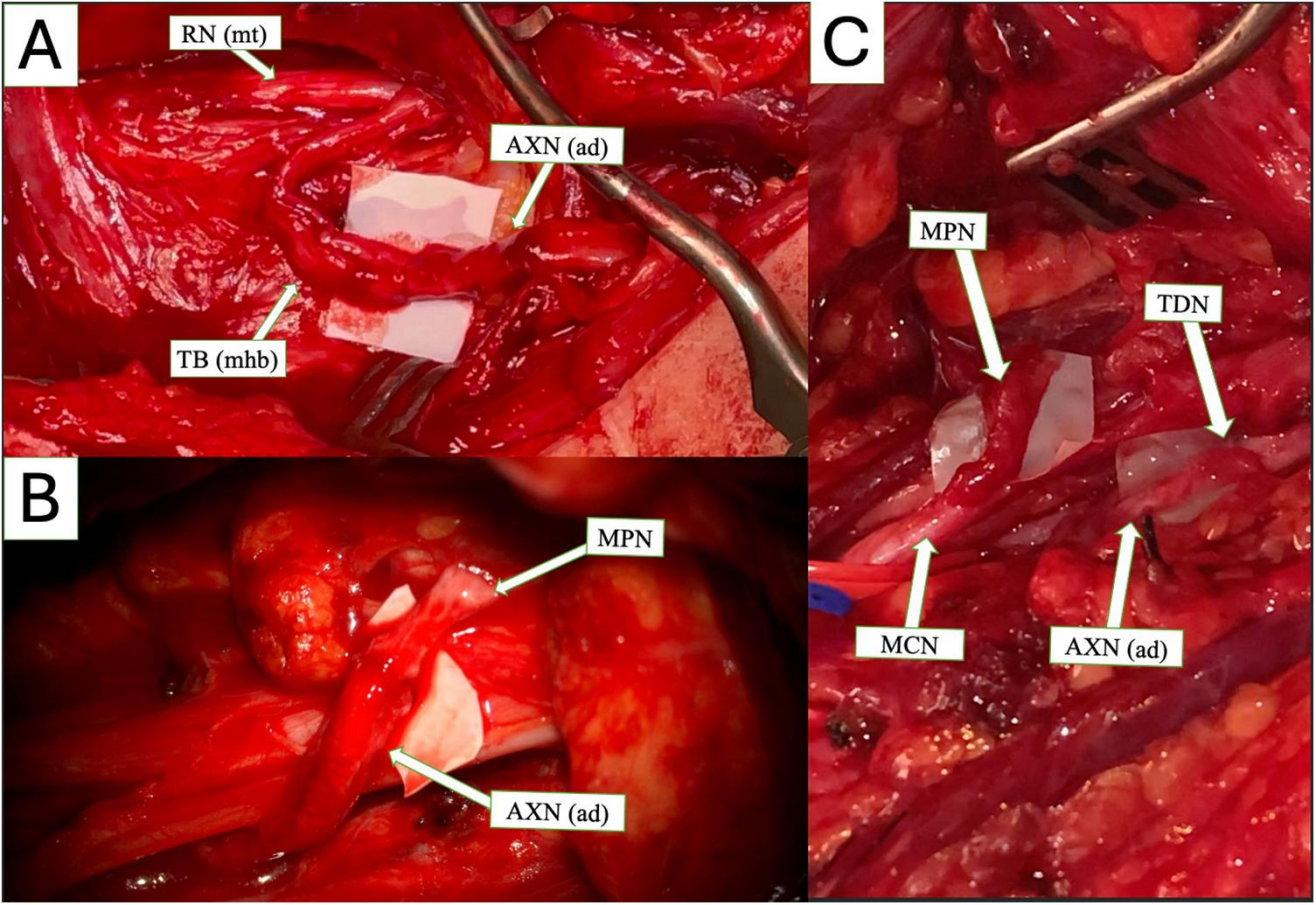
Distal nerve transfer techniques for shoulder abduction: **(A)** transfer of the radial nerve (RN) branch for the medial head (mhb) of the triceps brachii muscle (TB) to the axillary nerve (AXN) anterior division (ad) through the posterior approach; (*TBmhb-AXN*); **(B)** transfer of the medial pectoral nerve (MPN) to AXN (ad) through the anterior approach (*MPN-AXN*); **(C)** transfer of MPN to the musculocutaneous nerve (MCN) and the thoracodorsal nerve (TDN) to the AXN (ad) through the anterior approach (*MPN-MCN, TDN-AXN*).

**Figure 3:**
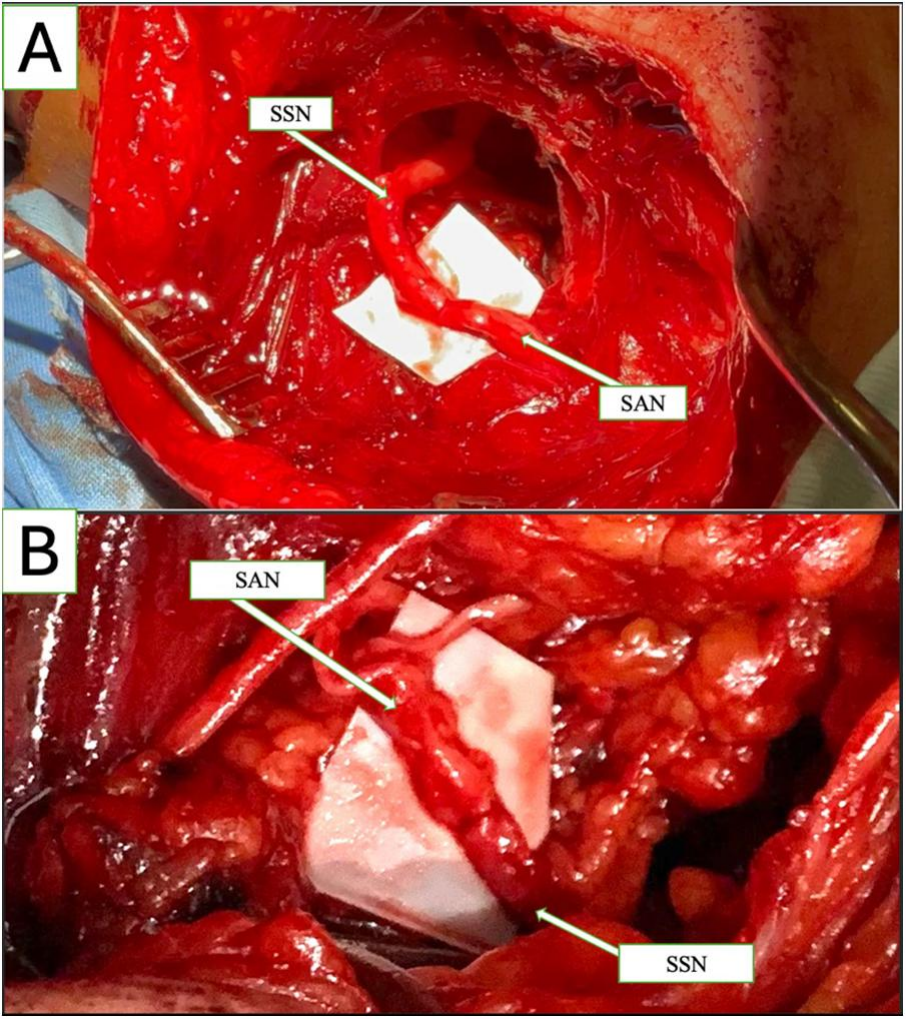
Distal nerve transfer for shoulder external rotation. **(A)** Spinal accessory nerve (SAN) to suprascapular nerve (SSN) transfer using the posterior approach. **(B)** SAN-SSN transfer using the anterior approach.

- In isolated AXN lesions, predominantly affecting shoulder abduction and flexion, and an RN branch to the medial or long head of the TB, transferred the anterior AXN division through a posterior approach (Figure 2) is considered the most feasible technique. This combination demonstrates high availability due to its constant anatomy and simple posterior approach, high suitability because of synergistic function, direct coaptation, and excellent axon match, and high acceptability given redundant TB innervation and negligible donor morbidity (74–78). The AXN posterior division, innervating the teres minor and containing sensory fascicles, is typically not targeted due to the risk of axonal dispersion and reduced effective motor input to the deltoid. In anatomical variants where the posterior division contributes substantially to deltoid function, it may be selected following intraoperative stimulation to confirm appropriate motor topography (79)
- In isolated SSN lesions, primarily affecting shoulder external rotation and induction of abduction, the most feasible donor is the SAN (Figure 3), which represents a motor-pure extraplexal donor with favorable anatomical proximity and a synergistic activation pattern (80). The posterior approach for SAN-to-SSN transfer (Figure 3A) offers distinct advantages over the anterior approach (Figure 3B), including preservation of upper trapezius function, the ability to perform a distal coaptation close to the target muscle, and improved visualization of the SSN at the suprascapular notch, which can identify double-crush lesions missed anteriorly. However, the anterior approach remains valuable when combined with supraclavicular exploration and reconstruction are needed, and it provides a greater proportion of SAN axons (81–88). In cases when the SAN is unavailable or reserved for other transfers, additional donors may be considered (TDN (89), DSN (90–92), C7 root fascicles for the PM (93, 94), LTN (95), ICNs (96), PHN (97), as well as PHN communicating branch to the C5 root.
- In isolated SAN injury, distal nerve transfer from the posterior division of the upper BP may provide trapezius reinnervation, with restoration of shoulder elevation and abduction, marked pain reduction, and good functional recovery. Mild residual scapular winging may persist, but overall outcomes are considered as favorable due to short reinnervation distance and donor-recipient synergy (98). In isolated SAN nerve injuries, the most feasible donor was reported to be a fascicle from the posterior division of the upper BP trunk, due to short reinnervation distance and donor-recipient synergy, while using DSN, MPN, LPN, and SSN were also considered (99, 100).
- In isolated LTN injury, TDN transfer has shown good functional outcomes with reliable recovery of serratus anterior function, reduction of scapular winging, and improvement in shoulder elevation (101–103). In cases of avulsion BP injuries where TDN is not viable, the usage of MPN (104) and ICN can be considered (105).
- In upper BP palsy, simultaneous innervation of the SSN and AXN by SAN and RN branches is the most common strategy, since both contribute to shoulder abduction and external rotation. Due to shorter regenerative distance and preferential reinnervation of the supraspinatus muscle (106–109) the impaired infraspinatus function may lead to insufficient external rotation (110). Therefore, targeted reinnervation of the SSN branch for the infraspinatus muscle (111, 112), or AXN branch for the teres minor muscle, may be considered to provide favorable external rotation (113). The branch for the medial TB head is more feasible for reaching the AXN trunk to incorporate the motor branch to the teres minor while excluding sensory components, thereby improving rotational outcomes. In addition, the usage of a branch for a long BB head is less acceptable due moribidiy associated with reduced shoulder stability (31).
- In upper extended BP palsies (C5-C7), when the RN branches are not viable, available collateral BP branches may be considered (MPN (Figure 2B) (114, 115), TDN (Figure 2C) (116), LSN (117, 118), and LTN (119). The viability of collateral BP branches depends on their origin and level of BP injury. In infraganglionic lesions, C5–C6 stumps are preserved, and TDN may remain viable if its origin is proximal to the lesion and not C7-dependent. In supraganglionic C5-C7 avulsions, MPN and LSN are most consistently preserved (120, 121). If collateral BP branches are prioritized elsewhere, and motor fascicles of MN and UN can be considered. These provide adequate axonal load and low morbidity but are non-synergistic, requiring cortical re-education (122). LTN reinnervation provides restoration of scapular stability and improved shoulder mechanics when combined with proximal nerve reconstruction, addressing the more complex biomechanical deficit of scapulothoracic dyskinesis in this injury pattern (103, 123). However, in the setting of a limited donor pool, the prioritization between AXN and SSN as primary targets remains controversial, while LTN is not considered as a concurrent option (124, 125).
- In complete BP (C5-T1) palsies, with supraganglionar avulsions, the extraplexal donors remain the only option (126), while donor and recipient prioritization remains controversial; Some authors favor SAN transfer to the SSN, given its dual contribution to both abduction and external rotation (127–129), with potential ICN-LTN to provide scapular rotation. Others advocate the use of AXN reinnervation, arguing that SSN-mediated abduction is limited and external rotation often remains insufficient. Additionally, unrecognized distal injury to the SAN may compromise outcomes (130).

#### 4.2.2 Elbow function

Restoration of elbow function addresses two complementary movements (flexion and extension) and presents a significant target in both BPI and cervical SCI, but with different priorities. Restoring elbow flexion is, in general, considered a top-priority upper extremity function, usually targeted in combination with restoration of shoulder abduction and external rotation in upper BPI. In complete BPI with a limited donor pool, elbow flexion is considered more prioritized than elbow extension and shoulder functions, while hand function is usually not even considered (52).

In SCI, elbow extension is more targeted than flexion because flexion is usually already present, while extension is critical for functional independence in a wheelchair user. Therefore, the definite selection of the target function and proper technique mostly depends on indication, injury severity, and reconstruction feasibility (70).

##### 4.2.2.1 Elbow flexion

**Figure 4:**
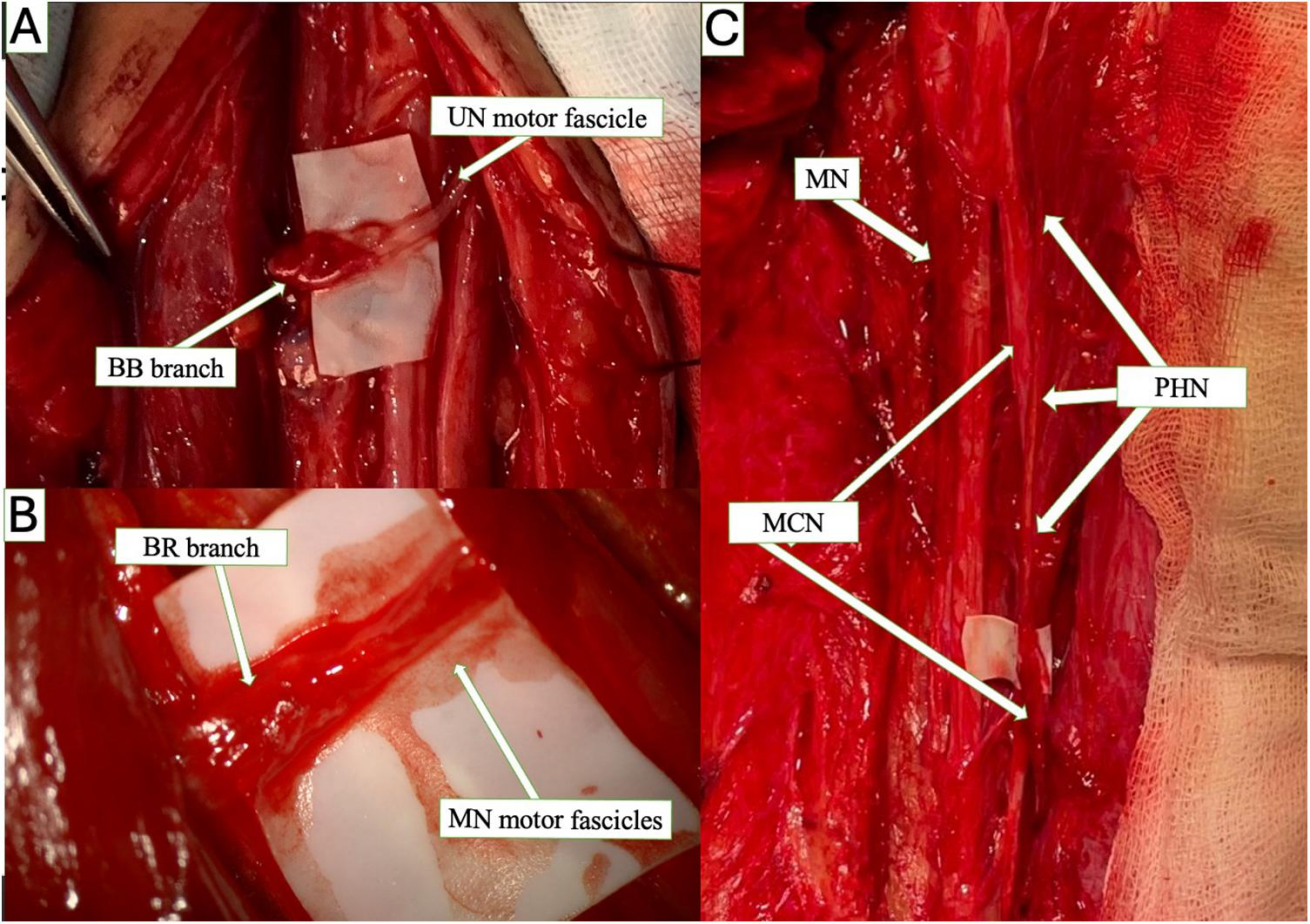
Distal nerve transfer for elbow flexion. **(A)** Ulnar nerve (UN) motor fascicle for flexor carpi ulnaris (FCU) to the musculocutaneous (MCN) branch for biceps brachii (BB); **(B)** Median nerve (MN) motor fascicle for flexor carpi radialis (FCR) to the MCN branch for brachioradialis (BR); **(C)** Phrenic nerve (PHN) transfer to the MCN branch for BB without using grafts.

Restoration of elbow flexion targets the MCN or its motor branches supplying the BB and, when necessary, the BR. (Figure 4) The BB branch is the preferred first recipient because of its larger size and easier dissection. Isolated transfer to the BR branch is less common but feasible when the BR branch is unavailable. Targeting the main trunk of the MCN is less common because it includes sensory fibers, which can dilute motor reinnervation; however, it may be necessary when distal branches are damaged (131–133).

**Figure 5:**
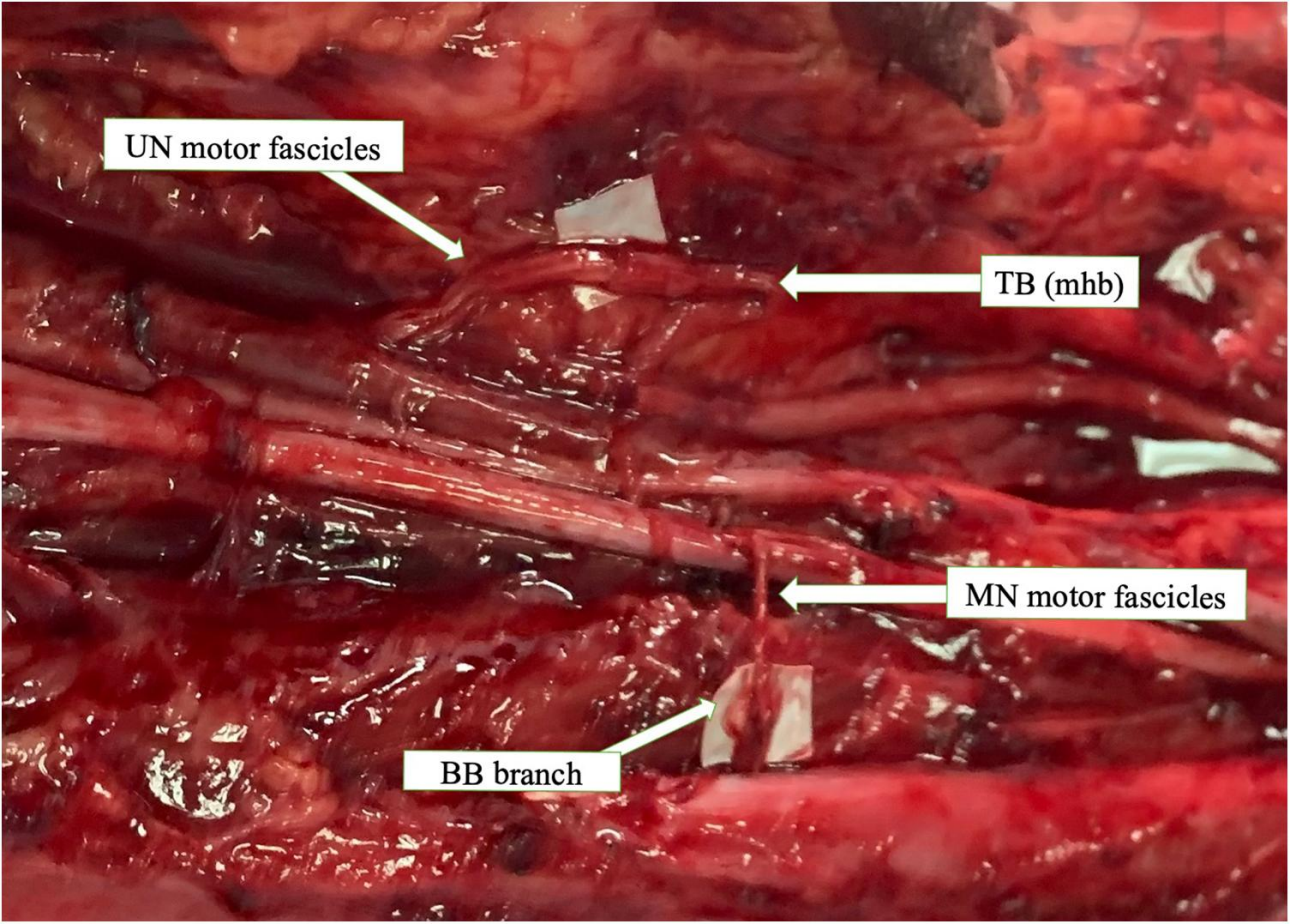
Distal nerve transfer for elbow flexion and extension in upper extended (C5-C7) brachial plexus (BP) injury. Ulnar nerve (UN) motor fascicles transferred to the branch for the medial head of triceps brachii (TB (mhb)); Median nerve (MN) motor fascicles transferred to the branch for biceps brachii (BB).

In isolated MCN and upper BP palsies (C5-C6), the most common technique is the Oberlin transfer (134–136) in which a single motor fascicle from the UN, typically supplying the FCU, is transferred to the MCN branch for BB. (Figure 4A) This procedure is highly reliable, providing direct end-to-end nerve coaptation, with sufficient axonal load and minimal donor morbidity. When greater axonal input is required, a double fascicular transfer may be performed by simultaneously adding another UN fascicle (modified Oberlin) (137) or an MN motor fascicle (Oberlin II) (138–140) to the MCN branch for the BR (Figure 4B). The most feasible MN fascicle is usually directed to the FCR, while the one for the FDS carries a risk of donor morbidity; the PT fascicle is less synergistic but still a highly feasible alternative (141). Both UN and MN are easily accessible in the upper arm and contain expendable fascicles. Despite a lower motor fiber proportion, the UN fascicle provides adequate axonal load for BB reinnervation. On the other hand, the MN fascicles contain a higher motor axon count, making them a strong alternative for BB reinnervation (142), particularly when UN must be reserved for other transfer options, such as in cases of extended (C5-C7) BP injuries (Figure 5).

**Figure 6:**
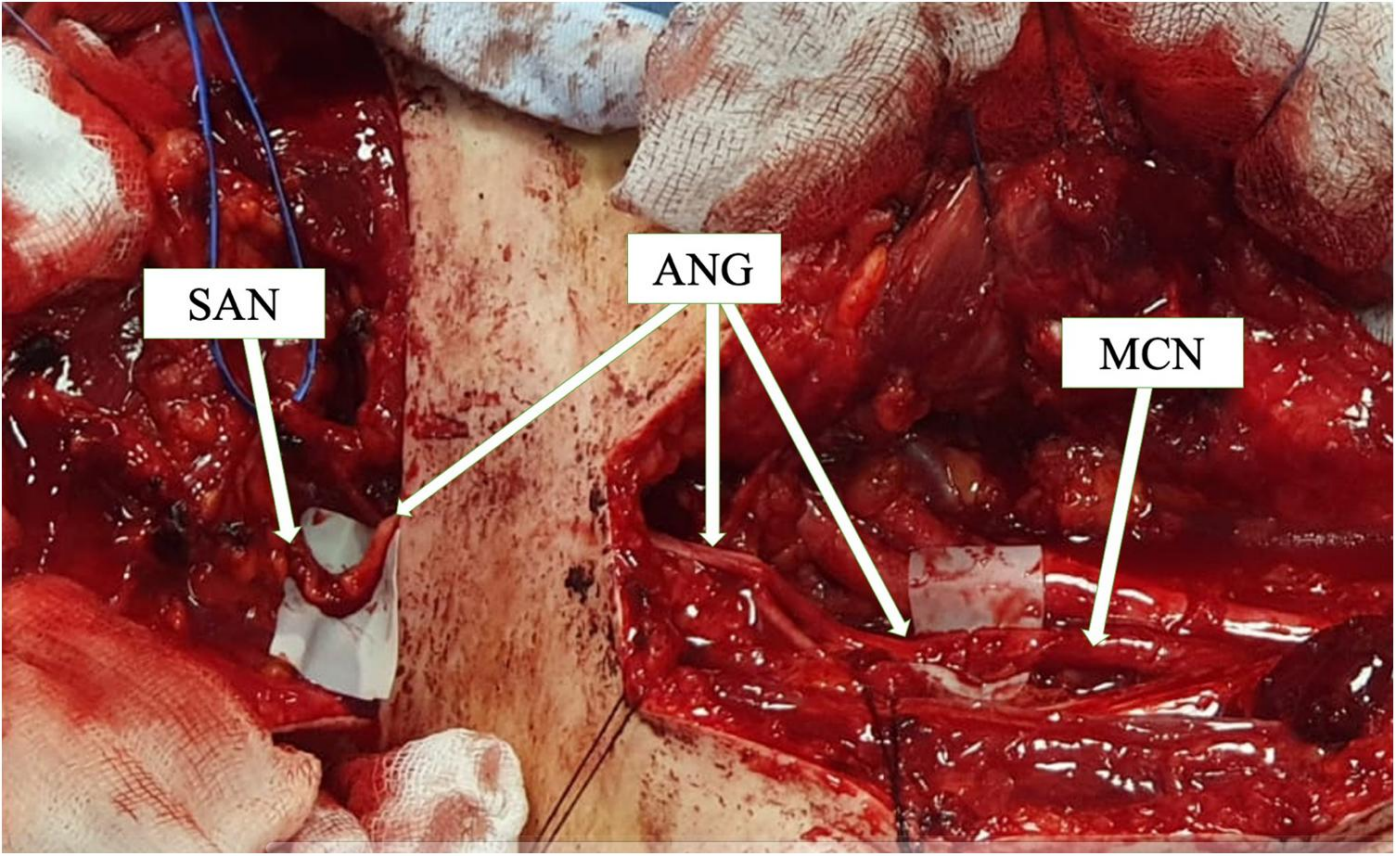
Spinal accessory nerve (SAN) to musculocutaneous nerve (MCN) transfer using autologous nerve graft (ANG) made of the cutaneous nerve of the arm.

In cases when the UN or MN fascicles are unavailable or reserved for other targets, the collateral BP branches or extraplexal donors can be considered, but present less feasible options, usually targeting the MCN as the recipient (132). The MPN (Figure 2C) offers moderate feasibility: it is pure motor with adequate axons, but often requires a graft due to insufficient length and diameter mismatch, while donor morbidity is minimal. The TDN has moderate feasibility when viable (C7 intact), but is rarely viable in C5-C7 injuries, and its synergy is poor. The LTN has low feasibility due to scapular winging and the need for graft repair (143, 144). The PHN has low to moderate feasibility; thoracoscopic harvest provides length (Figure 4C), but permanent diaphragm paralysis may reduce vital capacity. The SAN has low to moderate feasibility; it requires a long graft and is non-synergistic for elbow flexion. (Figure 6) The ICN offers low feasibility due to non-synergistic respiratory function, slower recovery, and acceptable donor morbidity, but remains a last-resort option in pan-plexus avulsions (145–148).

##### 4.2.2.2 Elbow extension

Reconstruction of elbow extension aims to reinnervate the RN branches supplying the TB, most commonly the long or medial head.

**Figure 7:**
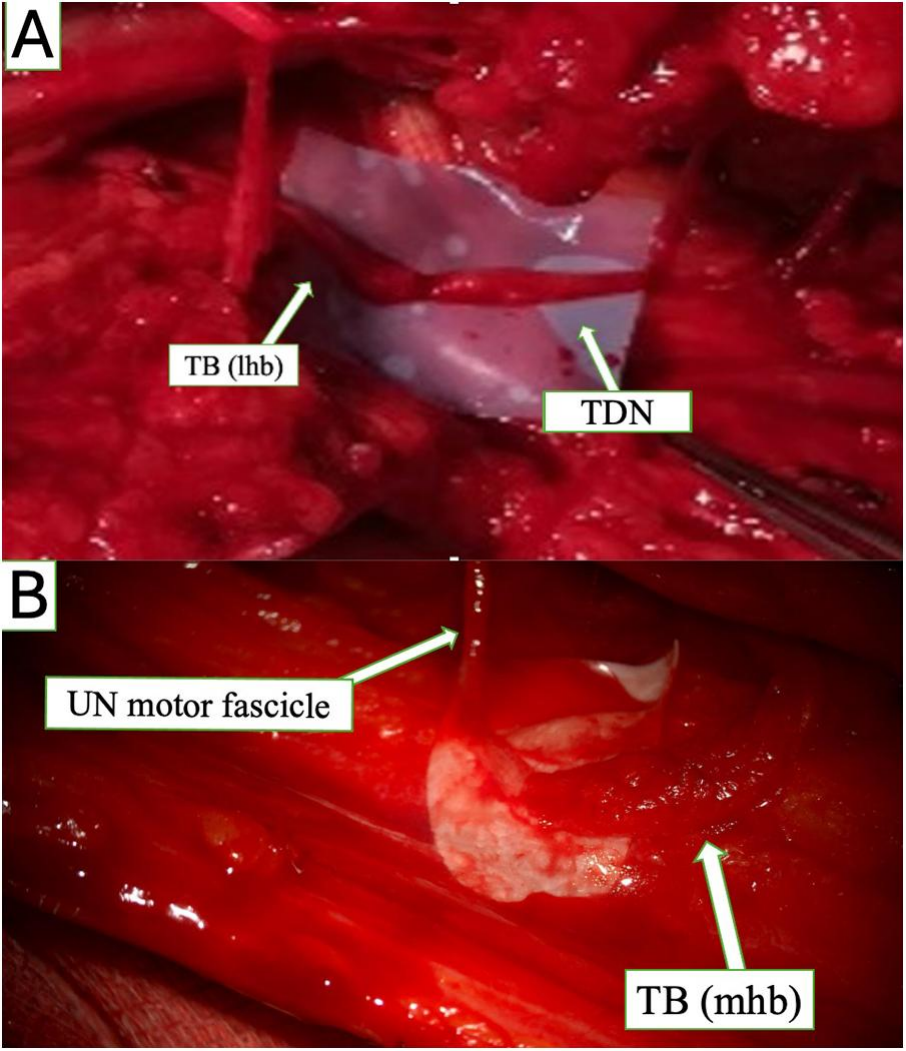
Distal nerve transfers for elbow extension. **(A)** Thoracodorsal nerve (TDN) transfer to the branch for the lateral head of triceps brachii (TB(lhb)). **(B)** Ulnar nerve (UN) motor fascicle for FCU to the medial head of triceps brachii (TB (mhb)).

- In isolated RN injuries, the TDN is the most frequently used donor due to its high content of strong motor axons and reliable anatomy. Usually, only one terminal branch is harvested to preserve partial latissimus dorsi function (Figure 7A). However, the TDN is often not viable in BP palsies, necessitating alternative donors (149).
- In upper extended BP injuries (C5-C7), the most commonly used donor is the fascicle of the UN to the FCU (Figure 7B) (150–154), while MN fascicles, DSN, MPN, and a non-triceps fascicle of the RN supplying wrist/finger extensors may also be used (155).
- In global BP avulsions, ICNs (156), PHN (157), and SAN (158) as donors are possible, with standard feasibility constraints (159).
- In cases of middle SCI (C5-C6) with preserved posterior DM function, the posterior division of the AXN may be transferred to the TB branch, providing a synergistic donor with no residual deficit (160). In same or lower level cases, with preserved elbow flexion, the MCN branches for BR can be transferred to the RN branches for TB (161, 162).

#### 4.2.3 Hand function

Hand function restoration via distal nerve transfers targets multiple independent functions: wrist/finger extension, wrist/finger flexion, thumb opposition, intrinsic hand function (claw correction, pinch), and tactile sensation. Each requires a specific donor-recipient pair tailored to the level and pattern of nerve injury.

##### 4.2.3.1 Wrist and finger extension

Reconstruction of wrist and finger extension focuses on reinnervation of the PIN for finger extension and the RN motor branch to the ECRB for wrist extension. These transfers are typically performed through a volar forearm approach that allows access to both donor and recipient nerves through a single incision. All donor-recipient combinations for wrist and finger extension demonstrate high feasibility, but their optimal use depends on the injury level and clinical context. No single combination is universally superior; selection should be guided by the injury pattern, donor availability, and specific functional goals (163).

**Figure 8:**
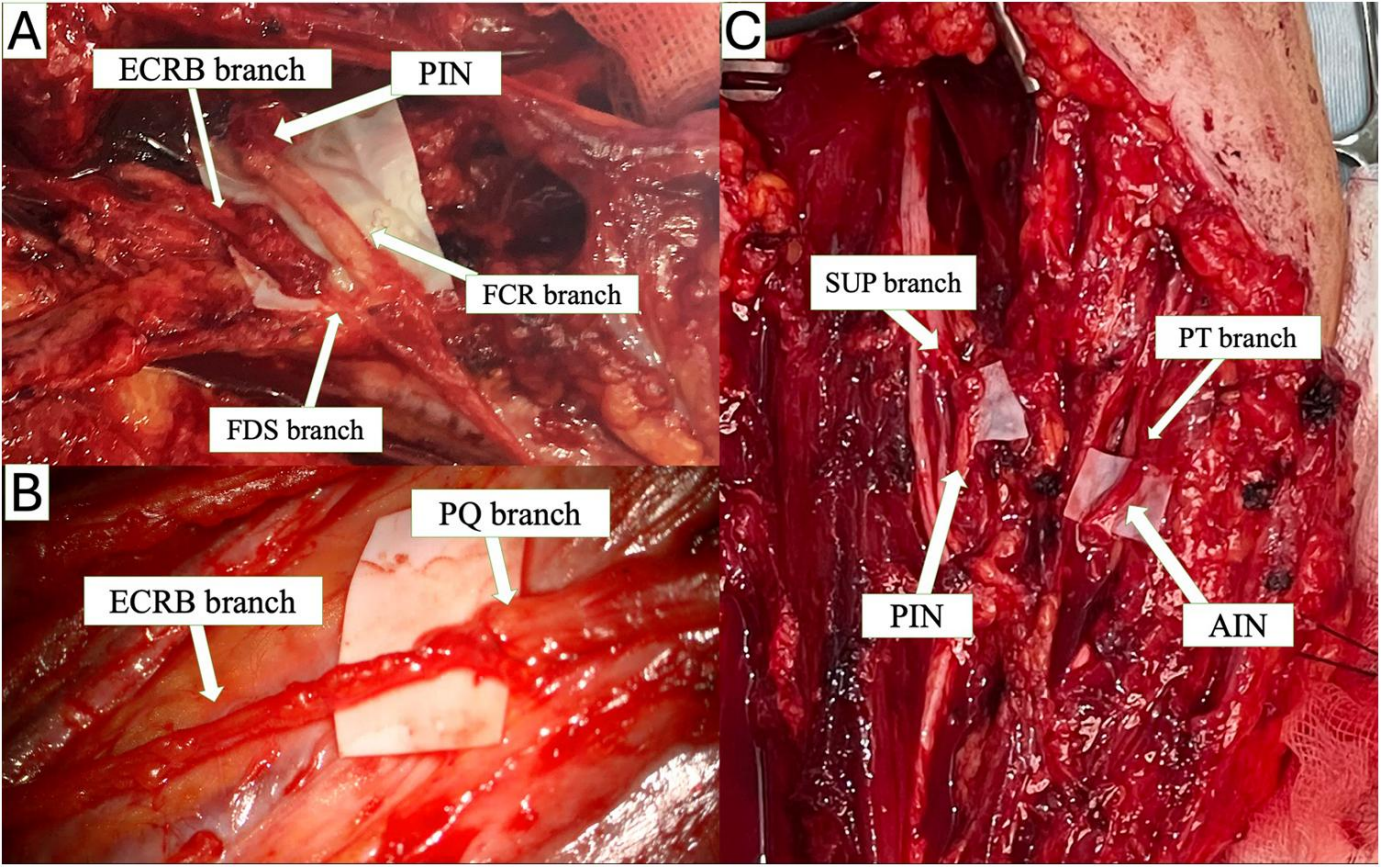
Distal nerve transfer techniques for wrist and finger extension. **(A)** Transfer of the flexor carpi radialis (FCR) branch to the posterior interosseus nerve (PIN) and the flexor digitorum superficialis (FDS) branch to the extensor carpi radialis brevis (ECRB) branch in an isolated high radial nerve lesion (FCR-PIN, FDS-ECRB); **(B)** Transfer of the anterior interosseus nerve (AIN) branch for pronator quadratus (PQ) to the ECRB branch in upper extended (C5-C7) brachial plexus (BP) palsy (PQ-ECRB). **(C)** Transfer of the supinator (SUP) branch to the PIN and pronator teres (PT) branch to the AIN in lower (C8-T1) BP injury (SUP-PIN, PT-AIN).

- In isolated proximal RN injuries (164), the most common combinations are the MN branch for FCR transferred to the PIN and the FDS branch transferred to the ECRB (Figure 8A). Both combinations offer direct coaptation, synergy, and minimal donor morbidity. The PT branches of the MN are a highly feasible alternative (165), and both distal and proximal can be transferred to the ECRL, ECRB, or PIN without using grafts. The FDS branch can also be transferred to the ECRL and PIN with high feasibility (166, 167).
- In upper extended BPI (C5-C7), the AIN branch for PQ may be transferred to the ECRB branch and restore wrist extension, providing a short regeneration distance, while preserving pronation via the PT function (168, 169) (Figure 8B).
- In lower BPI (C8-T1) RN branches to the SUP may be transferred to the PIN as highly feasible (Figure 8C), or directly to the smaller PIN branches: extensor carpi ulnaris (ECU), extensor digiti quinti (EDQ), and extensor digitorum communis (EDC) to provide selective reinnervation of extensor muscles (21, 170, 171).
- In selected cervical SCI patients with preserved elbow flexion, transfer of the BRC branch as a synergistic donor to ECRL or ECRB (172) or transfer of the branch for the SUP to the PIN (173) can be used to restore wrist extension.

##### 4.2.3.2 Wrist and finger flexion and thumb opposition

**Figure 9:**
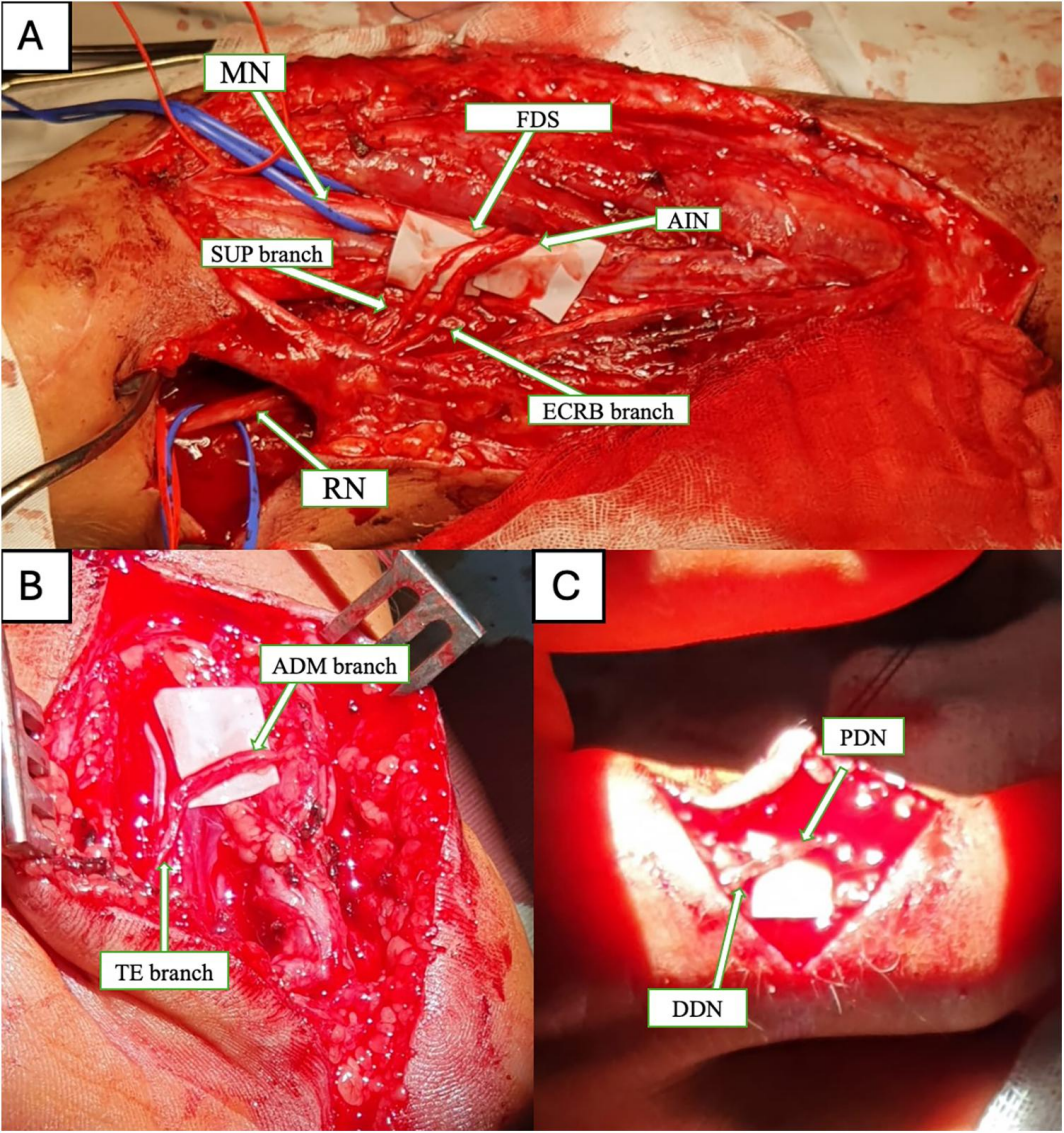
Distal nerve transfer for wrist and finger flexion, thumb opposition, and protective/tactile sensation. **(A)** Supinator branch (SUP) of the radial nerve (RN) transferred to the median nerve (MN) branch for the flexor digitorum superfitialis (FDS), and the extensor carpi radialis brevis branch (ECRB) of the RN transferred to the anterior interosseus nerve (AIN); **(B)** Ulnar nerve (UN) branch for adductor digiti minimi (ADM) transferred to the MN branch for thenar eminence (TE); **(C)** RN dorsal digital nerves (DDNs) transferred to MN palmar digital nerves (PDNs).

Restoration of wrist and finger flexion is performed by targeting the AIN and FDS branches of the MN (Figure 9). Thumb opposition is restored via the thenar (TE) motor branch of the MN. These transfers are typically indicated in isolated high MN injuries, lower BP palsies (C8-T1), isolated AIN lesions, and isolated thumb opposition deficits. All donor-recipient combinations demonstrate high to moderate feasibility, with selection guided by injury level, donor availability, and functional priorities.

**Figure 10:**
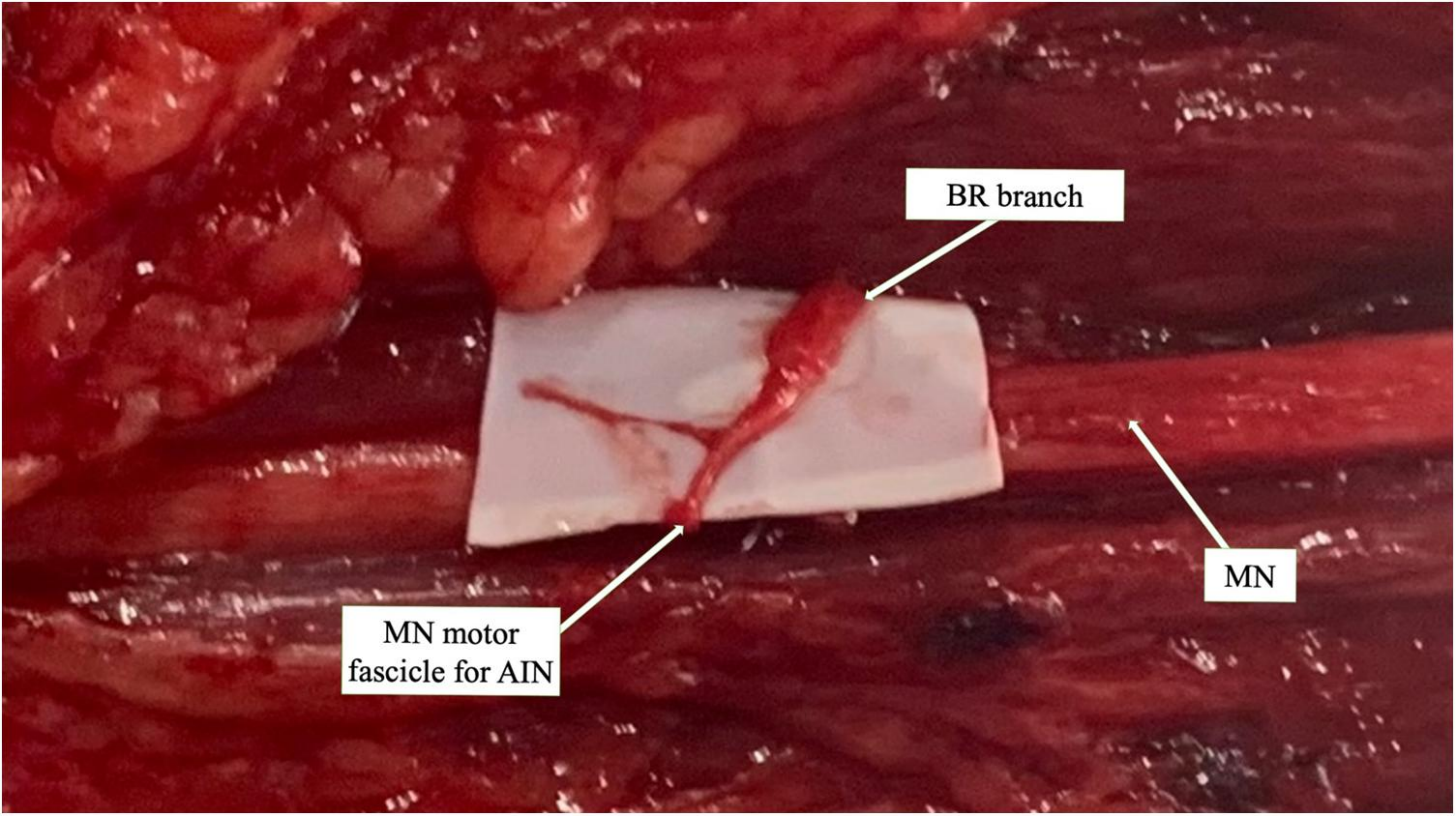
Distal nerve transfer in SCI (C6-C7)- the musculocutaneous nerve (MCN) branch for brachialis (BR) was transferred to the median nerve (MN) fascicle for the anterior interosseus nerve (AIN).

- For isolated AIN injury, the FDS or FCR branch of the MN transferred to the AIN is highly feasible: both donors lie in the same volar forearm field, allowing direct coaptation without graft, and are expendable, with the FCR offering superior length for tension-free repair (174).
- In isolated high MN injury (proximal to the AIN origin), the ECRB-AIN transfer is the most feasible option because it is synergistic (175), and wrist extension remains preserved via function of the ECRL and ECU, while additional SUP-FDS transfer complements it by enhancing proximal interphalangeal joint flexion (176) (Figure 9A). The BR-AIN transfer is less feasible due to the need for proximal MN neurolysis and a risk of aberrant reinnervation (177). For thumb opposition in isolated MN injuries, the ADM-TE transfer is the most feasible option (Figure 9B) due to direct coaptation, short-distance for regeneration, and minimal donor morbidity (178, 179). The alternative option, the PB-TE transfer, is less feasible but requires microdissection (180).
- In lower BP palsy (C8-T1) with the MN intact, the PT-AIN (Figure 8C) transfer is highly feasible because the PT and wrist/finger flexors are expendable and provide direct, synergistic coaptation; The BR-AIN remains a moderate second-line option.
- In a complete combined high MN and UN injury, only the ECRB-AIN transfer is feasible for finger flexion, while opposition and wrist flexion require tendon transfers.
- In tetraplegia or lower cervical SCI (C7-C8) with preserved wrist extension), the branches for BR (Figure 10); BRC, SUP, or ECRB can be transferred to the AIN (175, 181–184) or as a double transfer in combination with the transfer to the branch for FDP (185)
- In isolated thumb opposition deficit (TE) branch injury with intact UN), the ADM-TE transfer remains the gold standard, with the third lumbrical and FDMB branches directed to the reccurent MN branch also considered feasible (186). The UN branches for the FPB (present in only −50% of individuals) and PB provide only moderate feasibility due to variable anatomy or small nerve caliber (180).

Across all patterns, high-feasibility transfers are characterized by direct coaptation, short reinnervation distances, expendable donors, and minimal donor morbidity, whereas moderate-feasibility options are reserved for cases where first-line donors are unavailable.

##### 4.2.3.3 Intrinsic hand function

**Figure 11:**
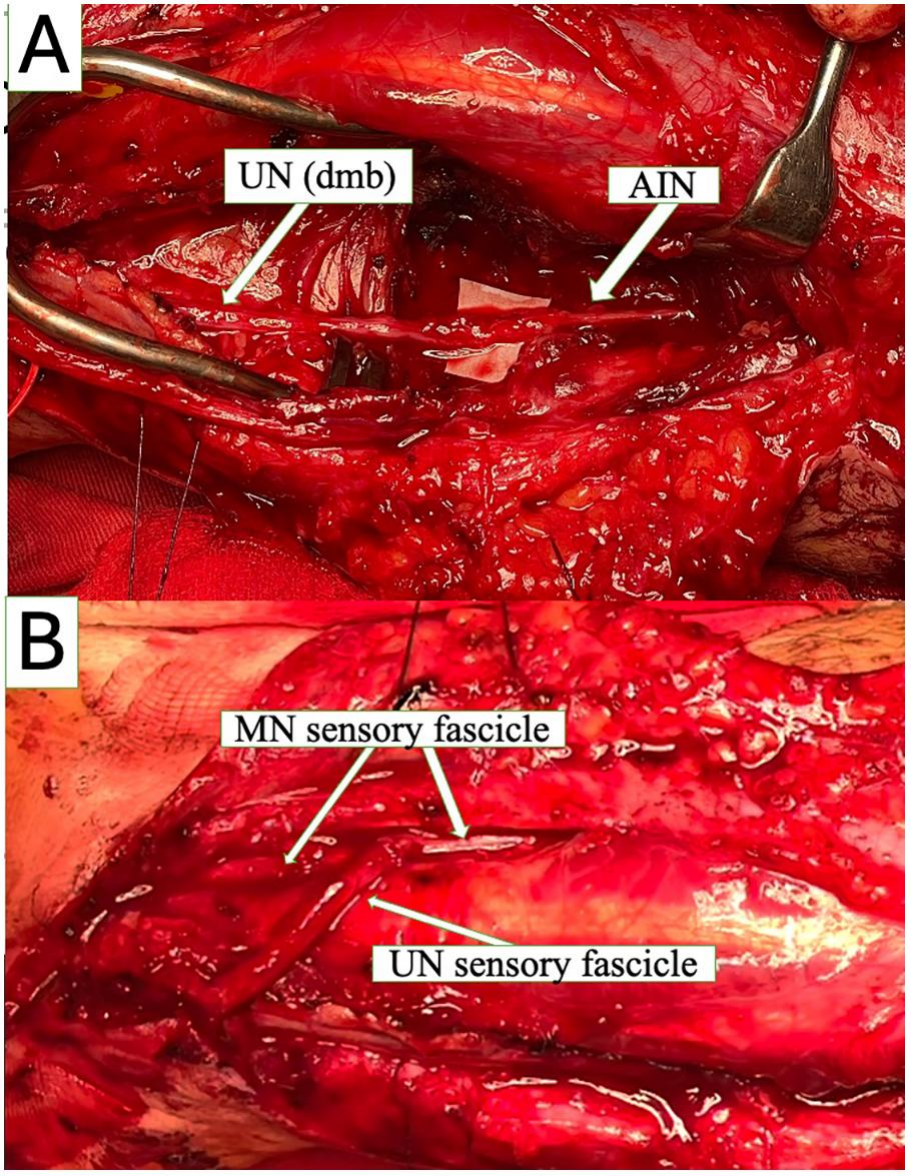
Distal nerve transfer for intrinsic hand function and protective sensation. **(A)** Transfer of the anterior interosseus nerve (AIN) distal branch for pronator quadratus (PQ) to the ulnar nerve (UN) deep motor branch (dbm); **(B)** Median nerve (MN) sensory fascicle transferred to UN sensory branch.

For intrinsic hand function restoration targeting the deep motor branch of the UN, the distal AIN-to-deep UN motor transfer has emerged as a rational strategy (Figure 11A) (187). The AIN branch to the PQ is considered most feasible donor because it is expendable (pronation preserved by the PT) and has adequate length and axon count for direct coaptation. Intraneural dissection of the UN is then performed to isolate the deep motor branch (188, 189). Alternative donors such as MN fascicles (FDS or FCR) are moderately feasible but may be needed for other reconstructions. PIN branches (EDM or ECU) have low feasibility due to long graft requirements and unfavorable axon counts and are not recommended as primary options (190–195).

##### 4.2.3.4 Protective sensation

Restoration of protective sensation in high MN and UN injuries is achieved through expendable distal sensory nerve transfers that provide rapid reinnervation of critical palmar surfaces with minimal donor morbidity. To restore sensation in high MN lesions when standard repair is not feasible, the dorsal digital nerves (DDNs) of the RN can be transferred directly to the palmar digital nerves (PDNs) of the thumb and index finger (Figure 9C). This technique offers a very short regeneration distance, uses expendable donors, and has high feasibility (196). To restore the protective sensation of the UN, two options are available. The lateral antebrachial cutaneous nerve (LACN) can be transferred to the sensory fascicle of the UN in the same procedure, a feasible adjunct with minimal donor morbidity (197). Alternatively, an MN sensory fascicle can be transferred end-to-end or end-to-side to the distal stump of the UN sensory branch, providing meaningful reinnervation of the ulnar digits (Figure 11B) (198). Donor site morbidity is limited to minor numbness in the donor territory and is well tolerated. All three sensory transfers have high feasibility and can be combined with motor transfers in a single stage.

#### 4.2.4 Lower limb function

Distal nerve transfers for lower limb reconstruction are less common than in the upper extremity but are increasingly used for specific indications, particularly foot drop, quadriceps weakness, and selected tibial nerve deficits.

Common peroneal nerve (CPN) palsy is the dominant lower extremity indication. The most frequently reported strategy involves transfer of an expendable tibial nerve (TN) motor branch, most commonly a soleus (SM) branch, to the deep peroneal nerve (DPN) branch to restore ankle dorsiflexion; this donor offers the shortest regeneration distance and the highest axon count among tibial branches. The lateral gastrocnemius branch and the flexor hallucis longus (FHL) branch are moderately feasible alternatives when the SM branch is unavailable (199).

For femoral nerve (FN) palsy, the most established distal transfer strategy involves the transfer of the obturator nerve (ON) branches to FN motor branches to restore quadriceps function. Sartorius motor branches are a highly feasible alternative donor, as they are expendable, superficial, and provide an excellent axon match (200).

In selected cases of high sciatic nerve (SN) injury, FN motor branches have been transferred to both CPN and TN in cadaveric feasibility studies, though clinical data remain limited (201).

TN motor deficits have been reported as targets for distal nerve transfer in selected lower extremity reconstructions; however, the literature remains heterogeneous with respect to techniques, indications, and outcomes (45). Compared with CPN and FN applications, TN transfers are less standardized and remain confined to highly selected cases.

For the restoration of protective sensation on the plantar aspect of the foot, the saphenous nerve can be transferred directly to the sural nerve, a highly feasible procedure with minimal donor morbidity (202).

### 4.3 Technical modification level

Within the technique-modification level, variations in distal nerve transfer procedures can be organized into four domains (biological, architectural, spatial, and microsurgical) that together provide a structured framework for optimizing reconstruction while preserving the primary functional objective (203) (Table 3).

**Table 3:** Technical modification level of distal nerve transfer procedures.

Domain	Subcategory/Parameter	Options/Variations	Purpose/Clinical Rationale
Biological	Fascicular number	Single-fascicle	Balance axonal yield against donor morbidity
		Multi-fascicle	
	Donor utilization	Partial	Determined by functional redundancy and acceptable deficit
		Complete	
	Nerve fiber composition	Motor-dominant	Optimize motor reinnervation; mixed used only when anatomical constraints limit selective harvest
		Mixed motor-sensory	
	Fascicle targeting method	Intraneural topography	Guide fascicle selection when internal organization is uncertain
		Intraoperative electrical stimulation	
	Donor synergy	Synergistic	Synergistic donors facilitate cortical reeducation and motor retraining; antagonistic used only when synergistic unavailable
		Antagonistic	
	Laterality	Ipsilateral (routine)	Ipsilateral standard; contralateral reserved for highly selective circumstances
		Contralateral (exceptional)	
Arhitectural	Connectivity pattern	One-to-one	Match axonal distribution to functional demand; one-to-one for isolated targets; one-to-many for related targets with sufficient capacity; many-to-one to increase axonal density for high-demand functions
		One-to-many	
		Many-to-one	
	Resource allocation strategy	Single critical function	Depends on complexity and extent of neurological deficit
		Distributed across multiple functions	
Spatial	Coaptation location	Distalized (preferred) Proximal (when required)	Distalized shortens regeneration distance; proximal used due to anatomical limitations or surgical exposure
	Fascicular orientation	Standard/Rotated/Reoriented	Improve motor topographic alignment between donor and recipient
	Tension at coaptation	Tension-free (principle)/Minimal controlled tension (if unavoidable)	Excessive tension compromises microvascular perfusion and impedes axonal growth
	Procedural timing	Single stage/Staged	Single stage when all targets safely addressed; staged to reduce operative burden, simplify execution, allow interim functional assessment
Microsurgical	Coaptation type	End-to-end (standard) End-to-side	End-to-end for complete loss of continuity; end-to-side for partial injuries preserving donor continuity; reverse for retrograde entry; supercharged to reinforce insufficient native regeneration
		Reverse end-to-side Supercharged end-to-side	
	Gap management	Direct coaptation (tension-free) Interpositional graft (autologous nerve processed allograft, conduit, scaffold)	Direct preferred; graft or conduit used when gap prevents tension-free repair
	Fixation technique	Epineurial	Epineurial for rapid alignment of small nerves;
			Perineurial for precise fascicular matching;
		Perineurial Group-fascicular	Group-fascicular for large nerves with complex internal organization
	Coaptation stabilization	Sutures alone	Balance mechanical stability with minimal tissue manipulation
		Fibrin glue alone Hybrid suture-glue	

### 4.4 Implementation of adjunctive strategies

Adjunctive therapies are frequently required to support distal nerve transfer-based reconstruction, particularly when anatomical constraints, prolonged denervation, or incomplete reinnervation limit the effectiveness of nerve transfer alone. These interventions do not alter the primary functional indication, but instead serve to enhance axonal regeneration, preserve musculoskeletal integrity, facilitate motor reeducation, or compensate for residual deficits. Adjunctive measures are selected based on integration, the selected target function, and the feasibility of available techniques and their modifications. They are integrated at appropriate stages of the reconstructive pathway to optimize overall functional outcomes (60, 204).

#### 4.4.1 Biological adjuncts

Biological interventions such as molecular, cell-based therapies, and gene expression modulation are primarily confined to regenerative phases, where their role is to enhance the intrinsic capacity for axonal growth and improve the local microenvironment for regeneration (205). In the context of nerve transfers, these modalities currently have no established clinical role and remain largely limited to preclinical investigation (206). While cellular and molecular strategies aim to reduce inflammation and promote regeneration, mostly in nerve defects, gene expression therapy (207) could be a major factor in optimizing mixed nerve coaptation feasibility (208), especially if combined with tubulation end-to-end coaptation repair (209) subjected to a personalized structural design strategy (210).

#### 4.4.2 Stimulation-based approach

Stimulation-based interventions can be used to enhance functional outcomes following nerve injury and repair by optimizing the biological and neuroplastic environment for regeneration. Overall, these stimulation-based neuromodulatory interventions remain in an early and emerging stage within peripheral nerve surgery, with no robust clinical evidence specifically supporting their efficacy in nerve transfer procedures (211, 212).

- Electrical stimulation is applied at the peripheral level, primarily to reduce denervation-induced muscle atrophy, preserve neuromuscular junction integrity, and maintain muscle contractile properties during the period of incomplete or absent reinnervation. In later stages, it may contribute to the strengthening of reinnervated muscles, although its effectiveness is highly dependent on timing and stimulation parameters (39, 213, 214).
- Transcranial stimulation, including transcranial electrical and magnetic stimulation techniques, acts at the central level by modulating cortical excitability and influencing motor network plasticity. Its proposed role is to enhance cortical reorganization and strengthen descending motor drive to reinnervated muscles. Unlike peripheral stimulation, it does not directly activate muscle or peripheral nerve structures (204, 215).
- Targeted postoperative stimulation protocols are applied once early reinnervation is detected to promote selective activation of reinnervated muscles and reinforce appropriate motor patterns. These interventions support cortical reorganization and motor relearning by linking voluntary intent with muscle activation. Their effectiveness depends on precise timing and integration into structured rehabilitation programs (216).

#### 4.4.3 Tendon transfers

**Figure 12:**
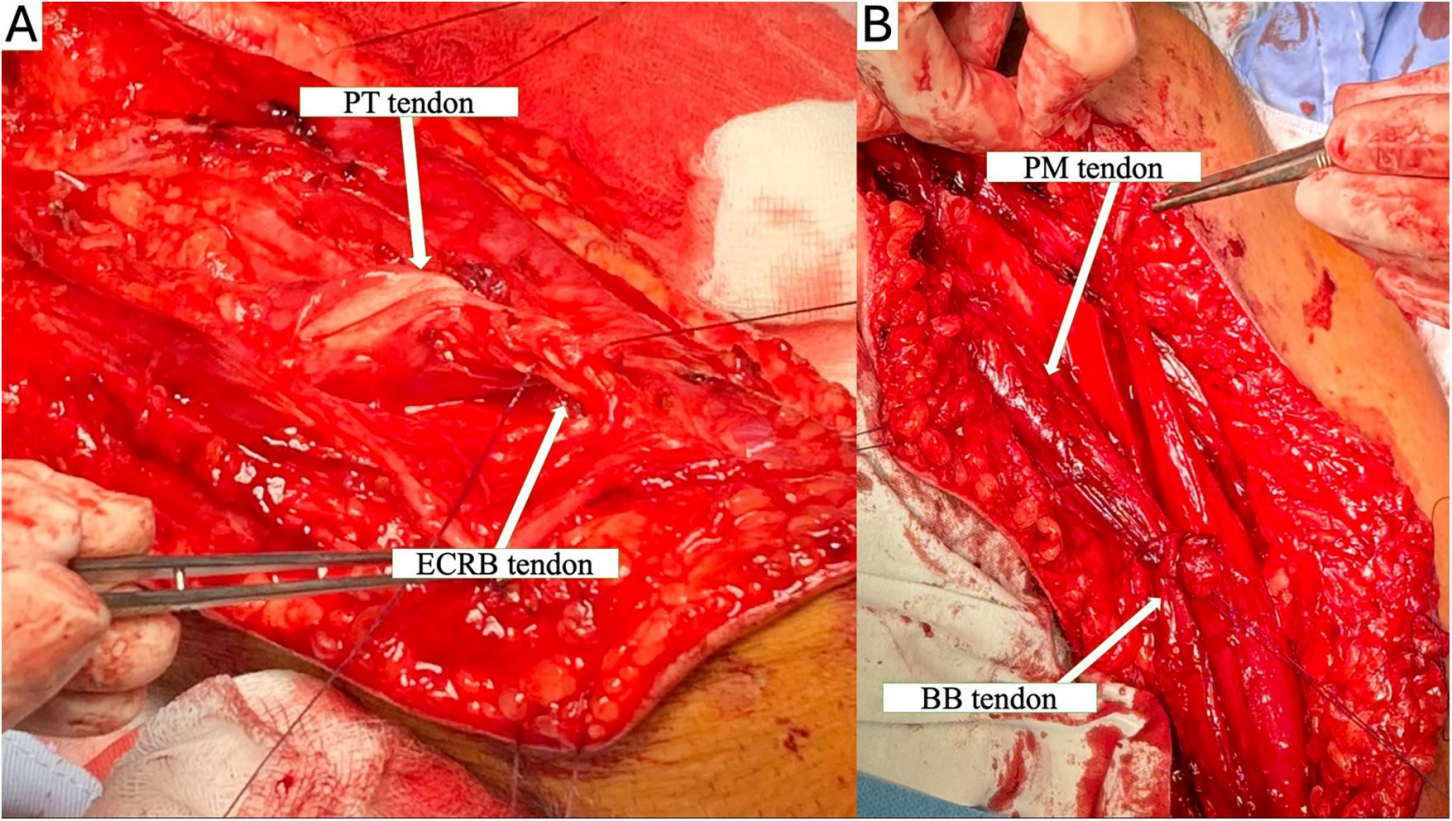
**(A)** pronator teres (PT) tendon to extensor carpi radialis brevis (ECRB) tendon transfer, performed simultaneously with distal nerve transfer for the radial nerve (RN) to facilitate wrist extension in long-standing lesions. **(B)** Pectoralis major muscle (PM) tendon transfer to the biceps brachii (BB) tendon to restore elbow flexion following failed repair of upper BP palsy.

Tendon transfers are compensatory rather than regenerative procedures and are typically used as an adjunctive modality in delayed presentations or when meaningful nerve recovery is unlikely (38, 59, 217, 218). They restore function by redirecting the force of intact musculotendinous units to substitute for lost motor activity, providing immediate mechanical recovery independent of axonal regeneration. However, they do not re-establish physiological motor control and have lower fatigue resistance compared with reinnervated muscle, limiting long-term functional performance. Accordingly, they are used selectively within reconstructive algorithms (41, 218). In chronic or irreparable cases, they function primarily as salvage interventions, while in selected patients with low expected recovery potential, they may be incorporated into planned biomechanical reconstruction. (Figure 12) This context-dependent role places tendon transfers at the interface between reconstruction and salvage, with classification determined by timing, indication, and expected biological recovery potential.

#### 4.4.4 Joint stabilization

Joint stabilization procedures are salvage interventions used in cases of severe, chronic, or irrecoverable motor deficit when nerve reconstruction and functional restoration are no longer feasible. These include arthrodesis, tenodesis, and other stabilizing techniques aimed at optimizing limb positioning, improving mechanical alignment, and enabling compensatory function in the absence of active motor control. By eliminating unstable or non-functional joint motion, these procedures enhance limb usability, improve force transmission from preserved musculotendinous units, and facilitate orthotic or assistive device use. However, they do not restore active movement or neuromuscular function and therefore represent definitive compensatory strategies within the salvage spectrum of peripheral nerve reconstruction (42, 219–221).

#### 4.4.5 Orthotic devices

In the acute and early postoperative period, immobilization reduces mechanical stress at the site of nerve repair or transfer, minimizes tension across coaptation sites, and helps maintain optimal anatomical alignment. It also contributes to the prevention of secondary complications such as joint instability and soft tissue contractures when appropriately positioned (222). As reinnervation emerges, orthotic device strategies may shift toward facilitating controlled motion and functional task performance. In patients with persistent weakness after brachial plexus injury, myoelectric devices such as the myoelectric elbow orthosis and powered variants have demonstrated improvements in elbow flexion strength, range of motion, and activities of daily living in small observational studies. More advanced strategies, such as the myoelectric orthosis bionic approach, describe adaptive control paradigms in which electromyographic input is reassigned from donor to reinnervated muscles over time; however, this is currently supported only by case-level evidence. Overall, orthotic management should be continuously adjusted to the patient's evolving neurological status, with a clear distinction between established protective functions and still-investigational assistive technologies, which remain insufficiently studied in the context of their application following nerve transfers (223–226).

#### 4.4.6 Prosthetic reconstruction

Amputation followed by prosthetic fitting represents a rare, last-resort adjunctive strategy in selected patients with global brachial plexus injury when nerve reconstruction, nerve transfers, and tendon transfers are unable to restore meaningful function. It is considered only after confirmation of absent or non-recoverable neurological potential. It is primarily aimed at improving mechanical function rather than alleviating pain, as neuropathic pain may persist or even be associated with phantom phenomena postoperatively. The procedure is indicated in cases of a heavy, non-functional limb with severe motor deficit and disabling deformity, where functional burden exceeds residual utility. Functional outcomes are optimized in patients with preserved scapulothoracic control, allowing effective use of modern myoelectric or bionic prostheses (227).

## 5 Clinical outcome patterns and limitations of current evidence

Outcome assessment in peripheral nerve surgery remains fragmented due to heterogeneous and often non-comparable scoring systems, inconsistent definitions of success, small cohorts, and limited integration of objective functional or electrodiagnostic measures with validated patient-reported outcomes. Nerve transfer outcomes are commonly assessed using diverse measures of motor strength, range of motion, pain, and functional performance, yet recovery rarely reflects the effect of a single procedure alone because most upper limb functions depend on coordinated activation of synergistic muscles with overlapping innervation. Consequently, identical transfers may produce different outcomes depending on whether the injury is isolated or part of a broader lesion pattern (228–232).

Functional recovery, therefore, reflects the cumulative effect of all reconstructed elements within a specific injury context, making direct comparison across lesion patterns methodologically limited and prone to bias (90, 96, 233). Nevertheless, despite substantial heterogeneity and methodological limitations, the available literature demonstrates outcome patterns broadly consistent with and partially supportive of the three testable predictions derived from our framework. These findings provide indirect support for the biological and clinical plausibility of the framework and justify prospective validation.

### 5.1 Prediction 1 (injury severity and reconstructive constraints)

The first prediction posits that increasing nerve injury severity, a higher number of required target functions, and progressively limited donor availability are associated with worse functional and patient-reported outcomes. The available literature generally demonstrates a graded decline in functional recovery from upper trunk to pan-plexus injuries, although confounding between injury extent and reconstructive options limits causal separation of individual determinants.

In shoulder reconstruction, functional outcomes depend on coordinated activation of the AXN, SSN, SAN, and LTN. Contrary to complete recovery in most treated patients with isolated injuries of these nerves (98, 101, 102, 104, 120, 121), clinical series indicate that when the same nerve is treated in combination with other associated injuries—such as in cases of combined AXN and SSN dysfunction—the outcomes individually decline and vary considerably across injury patterns and surgical strategies. While both abduction and external rotation are usually completely recovered in isolated AXN and SSN lesions, in upper BP palsy, external rotation is frequently less robust than abduction (74, 234, 235), though this finding is not uniform across all reports. In more extensive BP injuries, with reduced functionality of supporting synergistic muscles, increased functional targets for restoration, and limited donor availability, the outcomes of shoulder function further decline (120).

Relatively high rates of useful elbow flexion recovery are achieved in cases of upper BP lesions following established transfers, such as the Oberlin procedure (87, 236, 237). In C5-C7 lesions, outcomes become more variable, particularly for distal functions such as wrist and finger extension. In C8–T1 injuries, restoration of intrinsic hand function remains limited despite reconstruction attempts (171). In C5-T1 injuries, reconstruction typically prioritizes a limited set of functions, most commonly elbow flexion and partial shoulder stability, with overall outcomes generally inferior to those seen in upper trunk lesions. In cervical SCI (161, 238, 239), distal nerve transfers have enabled patients with preserved lower motor neurons to recover active finger flexion and extension, often achieving antigravity strength and improved grasp and release for activities of daily living, though independent thumb opposition and intrinsic hand function are rarely restored.

Across studies, greater injury severity is also associated with worse patient-reported outcomes, including DASH and SF-36 scores, although these associations are influenced by residual motor deficits and functional prioritization during reconstruction (228).

### 5.2 Prediction 2 (feasibility triad compliance)

The second prediction states that donor–recipient pairings satisfying all three feasibility criteria—availability, suitability, and acceptability—should produce better functional outcomes than pairings that violate one or more criteria. The strongest evidence for this prediction comes from shoulder reconstruction following BP injury, where multiple donors for the AXN have been compared.

A large series of 206 patients undergoing AXN reconstruction reported a clear donor hierarchy that directly mirrors the feasibility triad (120). The TB branch of the RN, which satisfies all three criteria—constant anatomy (availability), excellent motor axon match with direct synergistic coaptation (suitability), and redundant triceps innervation with negligible donor deficit (acceptability)—achieved the highest success rate. The transfer of the branch to the long head of the TB generally provides better shoulder abduction outcomes than the transfer of the medial TB branch, despite comparable technical feasibility (235). The long head branch is more synergistic with shoulder abduction and offers a shorter, more direct coaptation, illustrating that even minor differences in suitability affect outcomes.

The most successful donors following RN branches were LSN, also fully compliant, and the TDN, which is moderately compliant due to poorer synergy with shoulder abduction and its frequent unviability, but was still successful in most of the cases cases. In contrast, the less feasible donors, LTN and ICN, were unsuccessful in most cases. However, not all evidence across the literature is uniform. One meta-analysis (121) reported that ICN transfers achieved abduction strength comparable to the RN branches, with a less favorable range of motion. In another meta-analysis, when compared to ICNs, the UN and MN fascicular transfers provided no significant difference in BB muscle strength recovery, except that they provided shorter recovery period (240).

These discrepancies highlight that extraplexal donors may achieve similar motor strength at the cost of poorer biomechanical quality, reflecting a subtle violation of suitability due to non-synergistic firing patterns. Overall, a consistent gradient emerges: fully compliant donors achieve the highest success rates, partially compliant donors show intermediate results, and non-compliant donors show the poorest outcomes.

### 5.3 Prediction 3 (technical and adjunctive strategies)

The third prediction states that technical refinements and adjunctive modalities may improve overall outcomes, but they cannot compensate for inadequate donor nerve selection or the absence of a suitable donor. Evidence for this prediction is indirect but consistent across several domains.

#### 5.3.1 Technical modifications

According to meta-analysis studies, double fascicular transfer UN-BB and MN-BR yields higher rates of meaningful recovery than single fascicular UN-BB (241, 242). The double-transfer strategy (many to one), simultaneously incorporating TDN with RN branches for AXN reinnervation, demonstrates superior results to single transfers according to one study (116). A systematic review on nerve transfers in the lower extremity demonstrated that for CPN injury, the use of multiple donor branches to reinnervate the DPN achieved a good outcome in slightly more than half of patients, compared to when a single donor branch was used. In FN and ON injuries, all reported patients undergoing multiple branch transfers achieved a good outcome (45). Double innervation strategy (one to many), using SAN for both SSN and AXN, was also reported as favorable (237).

However, this benefit exists only when both donor fascicles are themselves feasible. According to a systematic review, when the UN is injured or unavailable, substituting it with a less suitable donor, such as ICNs, yields markedly lower success rates. However, regarding the ICNs, no significant differences were found when comparing the cases of direct repair with nerve grafting, and the selection of two rather than three or four ICNs for harvesting was shown to provide similar outcomes, with reduced risk of donor morbidity (243, 244).

The consensus on the superiority of nerve transfers over nerve grafts remains debatable. While one systematic study prioritizes nerve transfers over grafting for elbow flexion (245) the other concludes that there is no significant difference (246). The PHN transfer to the MCN branch for the BB was reported to provide satisfactory outcomes without significant difference, whether applied through grafting or direct repair, following thoracoscopic harvesting (247, 248).

The choice of anterior vs. posterior approach for SAN to SSN transfer has been debated. While one meta-analysis favored the posterior approach (249) another found no statistically significant difference (233). This suggests that when the same donor-recipient pair (SAN-SSN) satisfies the triad criteria, technical modifications do not fundamentally alter the outcome. Modified approaches using partial RN fascicles with end-to-side coaptation have been proposed to increase axonal load while minimizing donor morbidity (250).

In hand reconstruction, the AIN to deep UN motor transfer (end-to-end or SETS) has been associated with meaningful recovery of intrinsic function in multiple series (251, 252) However, attempts to use the PIN as a donor to the deep UN, which violates suitability due to long graft requirements and low axon count, have consistently failed to achieve comparable results (51), despite modifications in coaptation technique or graft material.

Across all domains, the literature supports the view that technical refinements such as increasing fascicular number or using many-to-one connectivity can optimize outcomes when a suitable donor is available. However, when the feasibility triad is violated, such as in cases when the donor is unavailable, requires a long graft, or causes major morbidity, no technical modification has been shown to fully compensate.

#### 5.3.2 Adjunctive strategies

Contrary to technical modifications, the tendon transfers are already established as a hybrid technique that can complement nerve transfers and enhance functional recovery, or serve as a salvage procedure in cases of failed nerve repair (45). However, it remains debatable whether they should become the treatment of priority in selected cases, such as in lower extremity nerve injuries.

- In RN injuries, two systematic reviews compared nerve and tendon transfer techniques, and both agreed that tendon transfer can achieve good functional results, while they disagreed on the magnitude of benefit and the associated risks. While one highlights higher failure rates for finger extension and a notable revision rate with tendon transfer (253), the other advocates tendon transfer as the preferred primary option (254). These discrepancies may reflect differences in inclusion criteria, outcome definitions, or patient populations. The choice between nerve and tendon transfer for RN, therefore, remains individualized, balancing the predictability and speed of tendon transfer against the theoretical advantage of restoring native muscle action with nerve transfer.
- In patients with tetraplegia, a systematic review study concluded that AXN to RN transfer achieves elbow extension strength comparable to traditional DM-TB tendon transfer but with no donor morbidity, supporting nerve transfer as a preferable first-line option when anatomically feasible (239).
- In patients with foot drop, tendon transfer for foot drop reliably improves patient mobility and satisfaction, regardless of surgical technique, and serves as an effective salvage procedure when nerve transfer is not indicated (255).

## 6 Discussion

Distal nerve transfer surgery, though increasingly applied over the past two decades, remains a relatively young and understudied field. The literature is characterized by an overwhelming diversity of injury patterns, donor nerves, recipient nerves, surgical techniques, and outcome metrics. This heterogeneity is not a flaw of individual studies but an inherent feature of the clinical reality: no two nerve injuries are identical, and the number of possible donor-recipient combinations far exceeds the evidence base that could ever be generated through prospective trials.

A central observation of this review is that the existing literature, often cited as “evidence-based” guidance, rests largely on cadaver feasibility studies, small retrospective case series, and systematic reviews that frequently contradict one another. For example, some meta-analyses find that double fascicular transfer for elbow flexion is superior to single Oberlin transfer (241), while others report no significant difference in ultimate strength (240). For AXN reconstruction, one large series reports a clear donor hierarchy of the RN branches for the TB (120), yet another meta-analysis finds comparable abduction strength between RN branches and ICNs as donors (121). For RN nerve palsy, two systematic reviews disagree on whether tendon transfer or nerve transfer should be first-line (253, 254). Even the choice of surgical approach for SAN-to-SSN transfer remains debated, with some studies favoring the posterior approach and others showing no difference (233, 249). The comparison between nerve grafting and nerve transfer is equally unsettled (245, 246).

These contradictions are not merely academic. They reflect fundamental problems in the field: small sample sizes, variable inclusion criteria, lack of standardized outcome measures, and publication bias. Systematic reviews themselves are susceptible to these biases, as they aggregate heterogeneous data without resolving the underlying variability. Moreover, patients with severe peripheral nerve injuries, especially BP palsy, are a minority of the world's population. No single center sees enough uniform cases to produce definitive evidence. Consequently, the literature cannot offer a reliable “menu” of best practices.

In this context, the present paper neither claims to solve the heterogeneity nor prescribes a fixed algorithm. Instead, we propose a four-level decision-making framework integrated with the feasibility triad (availability, suitability, acceptability) as a descriptive tool to map and articulate the heterogeneity inherent in distal nerve transfer surgery. Each component of the triad and each level of the framework contains multiple possible subcategories. The framework does not tell the surgeon which donor to choose; rather, it provides a structured language to describe why a particular choice was made, what constraints were present, and where the uncertainties lie.

Existing proposed strategies, such as Mackinnon's “donor distal” principle (24) or the various BP reconstruction algorithms (52, 256) offer useful heuristics but tend to assume that standard pairings are universally applicable. Our framework differs by placing feasibility as a dynamic, patient-specific, time-sensitive assessment at the foundation. It acknowledges that a transfer that is anatomically perfect on paper may be infeasible in a given patient due to prior surgery, radiation, body habitus, or donor nerve injury. It also explicitly incorporates donor acceptability, forcing consideration of functional trade-offs that are often underreported.

The three testable predictions derived from the framework are not intended to be proven by this paper but to guide future research. Prediction 1 (lesion severity gradient) is already widely observed; Prediction 2 (feasibility triad compliance) requires prospective studies directly comparing compliant vs. non-compliant pairings; Prediction 3 (modifications cannot compensate for poor donor selection) challenges the trend toward technical complexity as a substitute for sound donor choice.

We do not advocate abandoning the existing literature. Rather, we argue that surgeons should not blindly follow “guidelines” derived from low-quality or conflicting evidence. Instead, they should systematically assess availability, suitability, and acceptability for each patient, document their decisions transparently, and use the four-level framework to communicate their reasoning. Only through such structured, granular reporting can the field move beyond the current state of fragmented and often contradictory evidence.

## 7 Limitations

This paper is a Hypothesis and Theory article, not a systematic review. The framework has not been prospectively validated. The feasibility triad components are defined qualitatively; quantitative thresholds remain to be established. Finally, the framework applies primarily to motor nerve transfers; sensory and mixed nerve transfers may require additional considerations.

### 7.1 Methodological limitations of the framework

- The four levels of the framework are presented as sequential, but in practice, decision-making is often iterative and non-linear.
- The framework does not account for recursive feedback loops, such as when intraoperative findings force revision of earlier levels.
- The feasibility triad components are defined qualitatively but lack quantitative thresholds. For example, what constitutes “sufficient” axon count or “acceptable” donor morbidity varies by patient, injury, and surgeon; the framework does not resolve this subjectivity.
- The framework currently addresses motor nerve transfers almost exclusively. Sensory nerve transfers, mixed nerves, and specific applications such as facial reanimation or genital sensation restoration may require additional feasibility criteria not captured by the triad.

### 7.2 Limitations of the evidence base

- The framework's predictions rely on literature that is itself heterogeneous and of low-quality. Systematic reviews included in our analysis have variable inclusion criteria, risk of bias, and publication dates. The framework cannot correct these underlying evidential weaknesses.
- Most cited studies are retrospective, single-center, and lack blinded outcome assessment. The absence of prospective randomized trials for most donor-recipient comparisons means that the framework's predictions remain hypothesis-generating rather than hypothesis-confirming.

### 7.3 Limitations for clinical implementation

- The framework requires detailed knowledge of donor nerve anatomy, access to intraoperative nerve stimulation, and microsurgical expertise. It may not be applicable in low-resource settings where such tools are unavailable.
- The framework does not incorporate patient-specific factors beyond anatomy and feasibility, such as cognitive capacity for motor re-education, motivation, comorbidities, or socioeconomic support. These factors may significantly influence functional outcomes irrespective of technical feasibility.
- The framework assumes that the surgeon can accurately assess donor viability and recipient status preoperatively and intraoperatively. However, partial nerve injuries, axon counting, and the distinction between reversible neurapraxia and irreversible injury remain challenging even with current diagnostic tools.

### 7.4 Limitations related to outcome measurement

- The framework does not prescribe a specific outcome metric. As the literature shows wide variation in MRC grading, range of motion, patient-reported outcomes, and electrodiagnostic criteria, the framework cannot unify these disparate measures.
- The framework emphasizes motor recovery but does not address pain (neuropathic pain, neuroma formation) as a separate outcome or as a feasibility constraint. Donor nerve harvest may create new painful neuromas, which is not explicitly captured by the acceptability criterion.

### 7.5 Temporal and dynamic limitations

- The framework is static; it does not incorporate the possibility of late donor recruitment (nervew that became available after recovery of another injury) or staged reconstruction where donor feasibility changes over time.
- The framework does not account for plasticity limitations in older patients or those with central nervous system comorbidities, which may render a technically feasible transfer functionally unsuccessful.

## 8 Future directions

The four-level framework and feasibility triad proposed here are descriptive, not prescriptive. Their primary value is to provide a structured language for reporting and comparing distal nerve transfer procedures. To move the field beyond its current state of heterogeneous, often contradictory evidence, several priorities emerge.

- The framework requires prospective validation. Studies should be designed to test the three predictions explicitly: whether lesion severity and donor constraint (Prediction 1) predict outcomes in a graded fashion; whether triad-compliant donor-recipient pairings (Prediction 2) outperform non-compliant ones when injury severity is controlled; and whether technical modifications (Prediction 3) can rescue a fundamentally infeasible donor choice. Such studies would ideally be multi-center, with predefined feasibility criteria and standardized outcome measures.
- The field urgently needs consensus on core outcome sets for nerve transfer surgery. Currently, studies report MRC grades, range of motion, electrodiagnostic parameters, DASH scores, SF-36, and various disease-specific metrics without standardization. A minimum dataset should include: (a) donor and recipient nerve characteristics (axon count, diameter, synergy, coaptation type), (b) a standardized motor strength scale with clear definitions of “functional” recovery (e.g., MRC ≥3 for antigravity, MRC ≥4 for resistance), (c) donor-site morbidity (specific deficit, duration, impact on daily activities), and (d) patient-reported outcomes (pain, satisfaction, quality of life). Until such standardization is adopted, meta-analyses will continue to aggregate incomparable data.
- The role of adjunctive and salvage strategies, particularly tendon transfers, joint stabilization, and orthotic devices, should be better defined within the framework. Current evidence suggests that nerve transfers are preferable when donor feasibility allows, but tendon transfers remain valuable in delayed presentations or failed regeneration. Prospective registries comparing matched cohorts of nerve transfer vs. tendon transfer for the same indication would clarify their relative positions in the reconstructive algorithm.
- Technological advances may refine feasibility assessment. Intraoperative nerve stimulation and fascicular topography mapping can improve selection during fascicle harvesting. High-resolution ultrasound and diffusion tensor imaging may allow preoperative assessment of donor nerve quality and target muscle viability. Machine learning models could integrate multiple feasibility variables to predict individual patient outcomes, though such approaches require large, well-annotated datasets that do not yet exist.
- The framework should be extended to understudied domains. Lower extremity nerve transfers, sensory reconstruction, and applications in spinal cord injury (where lower motor neurons are preserved) remain supported by limited evidence. The same feasibility principles apply, but specific thresholds for axon count, regeneration distance, and acceptability may differ. International collaborative networks are needed to pool cases and generate meaningful data for these rare indications.

The field must acknowledge that definitive randomized trials for many donor-recipient pairings are impractical due to small patient numbers and injury heterogeneity. Registry-based observational studies with rigorous adjustment for confounders, including the feasibility triad variables, may be the most realistic path forward. The framework proposed here offers a common language for such registries, enabling comparison across centers and over time.

## 9 Summary

Distal nerve transfer surgery is characterized by substantial heterogeneity, conflicting evidence, and limited consensus regarding optimal reconstructive strategies. The proposed four-level framework integrated with the feasibility triad does not eliminate these limitations, but provides a structured conceptual model for analyzing donor selection, reconstructive prioritization, and technical decision-making. By shifting emphasis from rigid procedural algorithms toward explicit feasibility assessment, the framework may improve clinical reasoning, facilitate more meaningful comparison across heterogeneous studies, and support development of more standardized reconstructive strategies. Although distal nerve transfer surgery is unlikely to achieve the evidentiary consistency of high-volume orthopaedic procedures, systematic application of the feasibility triad, standardized outcome reporting, and collaborative multicenter data collection may substantially improve the quality of evidence and contribute to more reliable and patient-centered functional restoration.

## Data Availability

The original contributions presented in the study are included in the article/Supplementary Material, further inquiries can be directed to the corresponding author/s.
